# Global Expression Profiling of Transcription Factor Genes Provides New Insights into Pathogenicity and Stress Responses in the Rice Blast Fungus

**DOI:** 10.1371/journal.ppat.1003350

**Published:** 2013-06-06

**Authors:** Sook-Young Park, Jaeyoung Choi, Se-Eun Lim, Gir-Won Lee, Jongsun Park, Yang Kim, Sunghyung Kong, Se Ryun Kim, Hee-Sool Rho, Junhyun Jeon, Myung-Hwan Chi, Soonok Kim, Chang Hyun Khang, Seogchan Kang, Yong-Hwan Lee

**Affiliations:** 1 Department of Agricultural Biotechnology, Fungal Bioinformatics Laboratory, Center for Fungal Genetic Resources, and Center for Fungal Pathogenesis, Seoul National University, Seoul, Korea; 2 Center for Food and Bioconvergence, Seoul National University, Seoul, Korea; 3 National Institute of Biological Resources, Ministry of Environment, Incheon, Korea; 4 Department of Plant Biology, University of Georgia, Athens, Georgia, United States of America; 5 Department of Plant Pathology and Environmental Microbiology, Pennsylvania State University, University Park, Pennsylvania, United States of America; University of Toronto, Canada

## Abstract

Because most efforts to understand the molecular mechanisms underpinning fungal pathogenicity have focused on studying the function and role of individual genes, relatively little is known about how transcriptional machineries globally regulate and coordinate the expression of a large group of genes involved in pathogenesis. Using quantitative real-time PCR, we analyzed the expression patterns of 206 transcription factor (TF) genes in the rice blast fungus *Magnaporthe oryzae* under 32 conditions, including multiple infection-related developmental stages and various abiotic stresses. The resulting data, which are publicly available via an online platform, provided new insights into how these TFs are regulated and potentially work together to control cellular responses to a diverse array of stimuli. High degrees of differential TF expression were observed under the conditions tested. More than 50% of the 206 TF genes were up-regulated during conidiation and/or in conidia. Mutations in ten conidiation-specific TF genes caused defects in conidiation. Expression patterns *in planta* were similar to those under oxidative stress conditions. Mutants of *in planta* inducible genes not only exhibited sensitive to oxidative stress but also failed to infect rice. These experimental validations clearly demonstrated the value of TF expression patterns in predicting the function of individual TF genes. The regulatory network of TF genes revealed by this study provides a solid foundation for elucidating how *M. oryzae* regulates its pathogenesis, development, and stress responses.

## Author Summary

Rice blast disease, caused by *Magnaporthe oryzae*, destroys rice crop enough to feed 60 million people every year and has served as a model pathosystem for understanding host-parasite interactions. However, little is known about how *M. oryzae* globally regulates and coordinates its gene expression at the whole-genome scale. We analyzed the expression patterns of 206 *M. oryzae* genes encoding transcription factors (TFs) under 32 conditions, including infection-related developmental stages and various abiotic stresses, using quantitative real-time PCR. We focused on identifying the TF genes that are induced during the two most important infection-related morphogenetic changes; conidiation and infectious growth in rice. We identified 57 conidiation-specific TF genes and functionally characterized ten of them. Our data also showed that infectious growth *in planta* and oxidative stress responses *in vitro* involve largely overlapping groups of TFs. Comprehensive TF expression data and functional validation provided new insights into the regulatory mechanism underpinning pathogenicity and stress responses in *M. oryzae*. These data will also serve as a guide in studying the role of individual TF genes and the coordination of their expression in controlling development, pathogenicity, and abiotic stress responses in *M. oryzae*.

## Introduction

Fungal pathogenesis requires well-orchestrated regulation of multiple cellular and developmental processes in response to diverse stimuli from the host and the environment. Transcription factors (TFs) function as key regulators of such processes. Identification of TF genes, which typically represent 3–6% of the predicted genes in eukaryotic genomes, has been greatly facilitated by genome sequencing [1]. High-throughput methods for gene expression analysis have enabled studies on how TF genes are globally regulated under diverse conditions [2]–[4]. A combination of these approaches has uncovered putative roles and potential interactions of TFs in animals and plants [3], [5]. Although DNA microarrays have been successfully used to study global gene expression patterns, this approach may not be sensitive enough to accurately analyze low-abundance transcripts, including those from many TF genes [6]. Quantitative RT-PCR (qRT-PCR) has been shown to be five times more sensitive than microarrays [4], serving as an effective means for accurate quantification of TF transcripts.

The rice blast fungus *Magnaporthe oryzae*, one of the most devastating pathogens of rice and related grass species, undergoes sequential developmental changes to successfully infect host plants and complete the disease cycles. These processes include conidiogenesis, conidial germination, appressorium formation, penetration peg formation and infectious growth. Extensive studies have been performed to identify and characterize the genes that participate in these developmental changes and pathogenicity in *M. oryzae* [7]–[11]. Recent functional analyses of several *M. oryzae* TF genes demonstrated their critical roles in processes such as conidiation (*COS1*, *MoHOX2*, *MoHOX4*, and *COM1*; [12]–[14]), appressorium formation (*MoHOX7*, *MoLDB1*, and *Con7p*; [12], [15], [16]), infectious growth (*Mig1*, *Mstu1*, *MoHOX8*, and *MoMCM1*; [12], [17]–[19]), oxidative stress (*Moatf1*; [20]), and light regulation (*Mgwc-1*; [21]). However, how *M. oryzae* TF genes are globally regulated and coordinated at the transcriptional level has not been studied. To address this knowledge gap, we analyzed expression patterns of 206 TF genes under 32 conditions, including infection-related developmental stages and various abiotic stresses, using qRT-PCR.

To test the utility of expression profiles for predicting the role of individual TF genes in development and pathogenicity, mutants of selected TF genes were characterized. The resulting data clearly demonstrated their value. All the data from this study are publicly available through the Fungal Transcription Factor Database (http://ftfd.snu.ac.kr/magnaporthe), an online platform designed to systematically identify and catalog TF genes in fungi [22].

## Results

### Transcription factors in *M. oryzae*

The data extraction pipeline of FTFD identified 495 putative TF genes (4.5% of the 11,054 protein-coding genes in *M. oryzae*) using the InterPro terms associated with DNA-binding motifs. The proportion of TF genes in the total proteome of 23 other fungal and Oomycetes species ranged from 2.4% (*Laccaria bicolor*) to 6.4% (*Rhizopus oryzae*) (Table S1). Interestingly, 26 genes (5.3% of the TF genes) belonging to 9 different TF families appeared to be *M. oryzae*–specific based on the lack of orthologs in other species, which was determined using basic local alignment search tool (E<10^−50^) and InParanoid algorithm [23] (Table S2).

**Figure 1:**
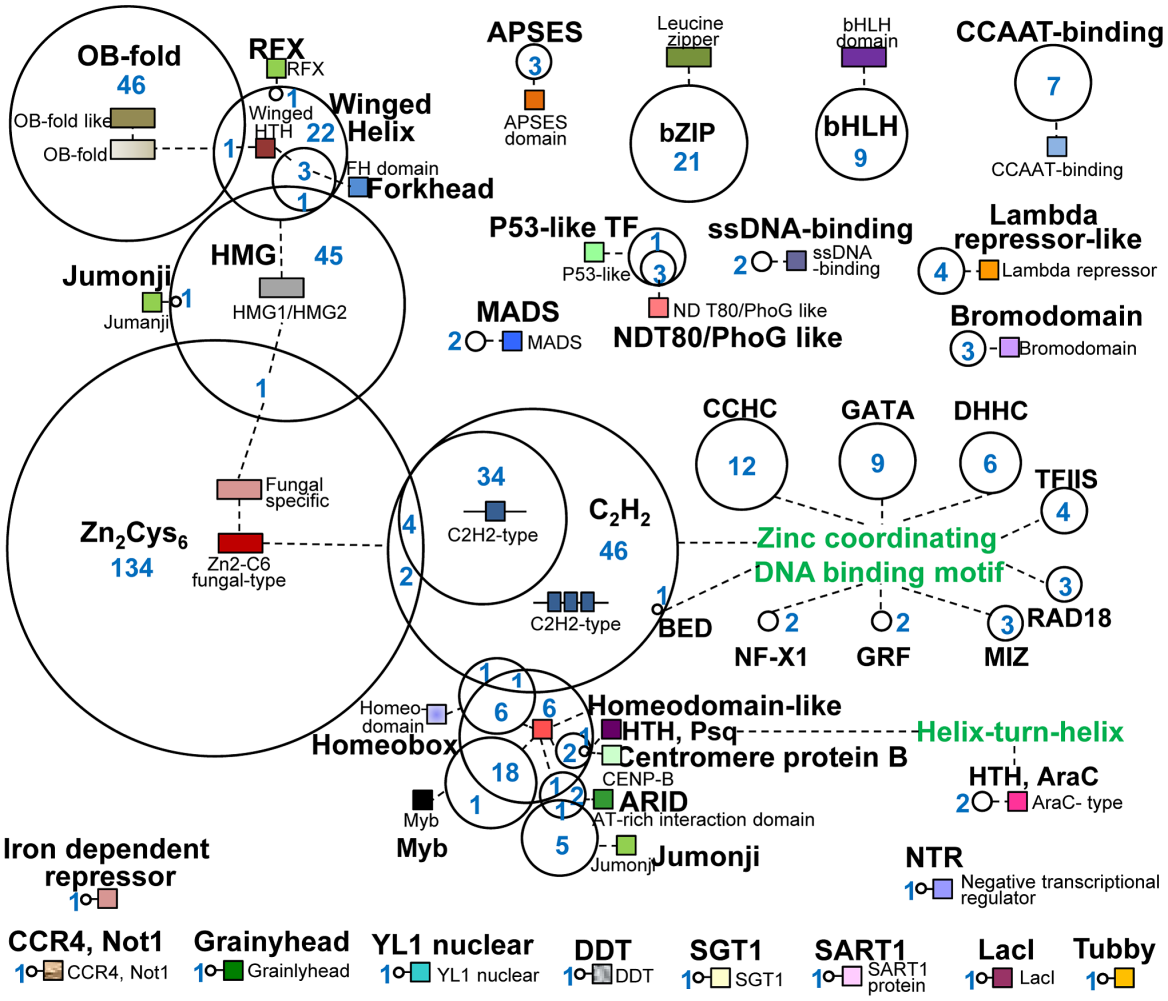
Identification and classification of 495 *M.* *oryzae* TF genes. These TF genes were identified based on a domain search using InterProScan (www.ebi.ac.kr/interpro) and belong to 44 families. The TF families are indicated by circles. Domains are noted by small colored rectangles. The TF genes at the overlapping regions between families indicate those possessing more than one TF motif. Two green-colored letters designate the zinc-coordinating DNA-binding motif and helix-turn-helix motif. The blue-colored number (in the circle or at the left side of the circle) indicates the number of genes in each family.

According to the InterPro classification [24], 495 *M. oryzae* TF genes were grouped into 44 families with the following four families dominating (Figure 1): fungal-specific Zn_2_Cys_6_ (141 genes; 28.5%), C_2_H_2_ zinc finger (89 genes; 18.0%), HMG (48 genes; 9.7%), and OB-fold (47 genes: 9.5%). Furthermore, 49 genes possessed more than one DNA-binding domains; among these, 29 of 35 homeodomain-like TF genes belonged to six different families. TFs with multiple DNA-binding domains are not unique to *M. oryzae* and have been detected in animals and plants [1], [25].

### Selection of reference genes for qRT-PCR

A few genes, such as tubulins, actins, and elongation factors, have been used as references for normalizing *M. oryzae* gene expression data generated using RT-PCR or qRT-PCR [12], [20], [26]–[32]. To identify the most stable reference gene under all the conditions used in our study, we evaluated seven candidate genes: *β-tubulin* [12], [31], [32], *actin2* [20], [29], [30], glyceraldehydes-3-phosphate dehydrogenase (*GAPDH*) [28], cyclophilin (*CYP1*) [26], [27], elongation factor1*β* (*EF1β*), *α-tubulin*, and ubiquitin extension protein (*UEP1*) (Table S3).

One of the widely used methods for identifying stably expressed genes is to calculate the cycle threshold (Ct). These seven genes showed a relatively narrow range of Ct mean values across all conditions (Figure S1A and B). To evaluate the stability of gene expression, we employed the GeNorm software [33]. Under all conditions tested, these candidate genes exhibited a high degree of expression stability with relatively low M values (less than 0.1), which are far below the default limit of M≤0.15 [33] (Figure S2A). For all samples, the most stable gene was *β-tubulin* with M value of 0.049, indicating that *β-tubulin* can be used as a stable reference gene under multiple conditions (Figure S2 B). To further validate the results obtained using GeNorm, we also employed Normfinder [34] and BestKeeper [35], which showed almost identical patterns (data not shown).

### Clustering of 206 TF genes based on their expression patterns under 32 conditions

We analyzed the expression patterns of 206 *M. oryzae* TF genes at multiple developmental stages and under various stress conditions that *M. oryzae* likely encounters during infection of host plants. These genes were chosen mainly based on their predicted significance and belong to 10 families, including one dominant and well-conserved family in fungi, plants, and animals (Zinc finger proteins [36]), two fungal specific families (Zn_2_Cys_6_ and APSES [37]), and those that are known to be involved in development (Homeobox [12] and bHLH [38]), cell differentiation (Myb [39]), and cell cycle (Forkhead [25]) (Table 1). The conditions analyzed included: (A) three developmental stages (conidiation, conidial germination, and appressorium formation); (B) two *in planta* infection stages at 78 hours post inoculation (hpi) and 150 hpi; and (C) 26 abiotic stress conditions (Table S4).

**Table 1:** The type and number of TF genes analyzed in this study.

Family	The number of TF genes analyzed
Zinc finger, Zn_2_Cys_6_	89
Zinc finger, C_2_H_2_	69
Zinc finger, GATA-type	7
Zinc finger, Rad18-type	1
HMG	2
Myb	17
Homeobox	8
bHLH	7
Forkhead	3
APSES	3
Total	206

The quality of RNA samples was evaluated using two pathogenicity genes with well- known expression patterns. The expression patterns of *MPG1* [40], a developmentally regulated gene, and *DES1* [26], which is up-regulated in the early stage of infection and under H_2_O_2_ stress, were consistent with published data (Figure S3A and B). We analyzed the abundance of transcripts of 206 TF genes under 32 conditions, and fold changes relative to levels in vegetatively grown mycelia were calculated using the 2^−ΔΔCt^ method [41].

**Figure 2:**
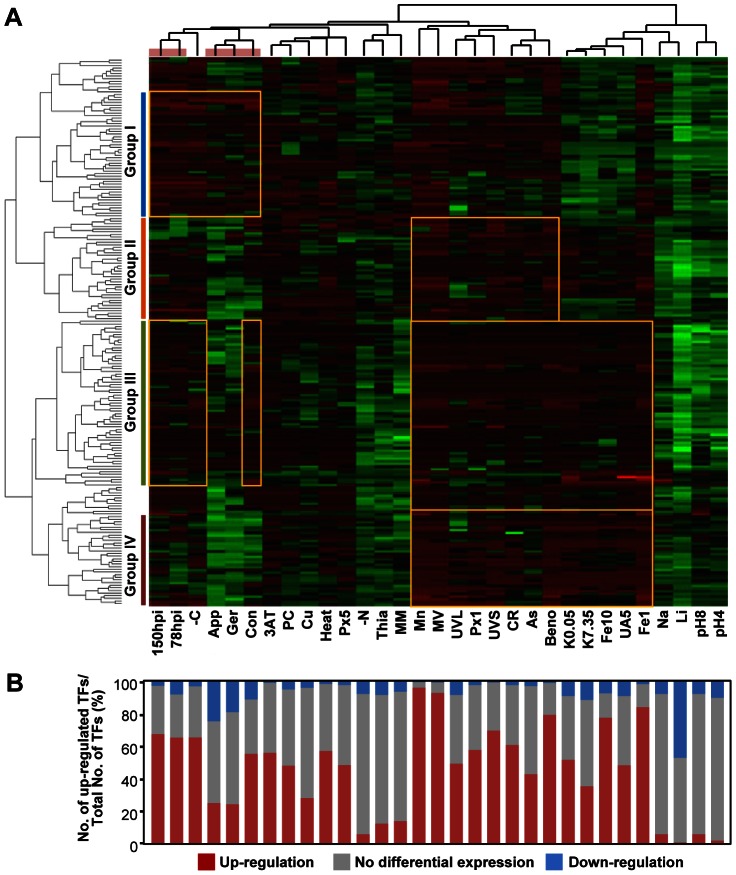
Expression profiles of TF genes. (A) Heat map showing expression patterns of 206 TF genes under 31 different conditions. The color for each gene indicates its expression level relative to its mean across all of the experiments. Red indicates up-regulation; black, no differential expression; green, down-regulation. Top, condition tree; left, gene tree. The pink bar at the top indicates five infection-related conditions. The yellow rectangles indicate distinct expression patterns in each group. (B) Percentages of up-regulated, not differentially expressed, and down-regulated TF genes under each condition.

Through a hierarchical clustering based on gene expression patterns, 185 of 206 TF genes were categorized into 4 groups with distinct expression patterns (Figure 2A). Group I contained 47 genes that were up-regulated preferentially at all infection-related developmental stages and under carbon (C)-starvation conditions and included the previously characterized TF gene *MoHOX7*, which regulates appressorium formation [12]. Genes in Group II (39), including *Mgwc-1* [21], *MoCRZ1* [9], and *Mstu1* [18] were induced preferentially by abiotic stresses. Group III contained 63 genes that were activated mainly at 78 and 150 hpi and under C-starvation and abiotic stresses caused by methyl viologen, H_2_O_2_, MnCl_2_, Congo red, FeSO_4_, and uric acid. None of the TF genes in this group have been characterized. Group IV consisted of 36 genes that were up-regulated by abiotic stresses, but not during 3 developmental stages, and included *COS1* [14] and *MoHOX1* [12].

The number of TF genes with significantly altered expression varied widely depending on the conditions (Figure 2B). Most TF genes were up-regulated (>2-fold) in response to treatment with methyl viologen (191, 92.7%) and H_2_O_2_ (119, 57.8%). More than 50% of the TF genes were up-regulated during conidiation and/or in conidia (112, 54.4%), host infection at 78 hpi (139, 67.5%) and 150 hpi (141, 68.4%). In contrast, less than 20% of the TF genes were induced in response to changes in nutrient conditions (i.e., minimal medium, nitrogen starvation, and thiamine treatment) and pH (4 and 8). Under ionic stress, MnCl_2_ induced the expression of most genes, whereas LiCl caused the down-regulation of the 47.3% of the genes (Figure 2B). Less than 20% of the genes were down-regulated in most of the conditions tested, except conidial germination (43, 20.8%), appressorium formation (54, 26.1%), LiCl (100, 48.3%), and 4 min UV irradiation (103, 49.8%) (Figure 2B).

### Expression profiles of the TF genes during infection-related developmental stages

**Figure 3:**
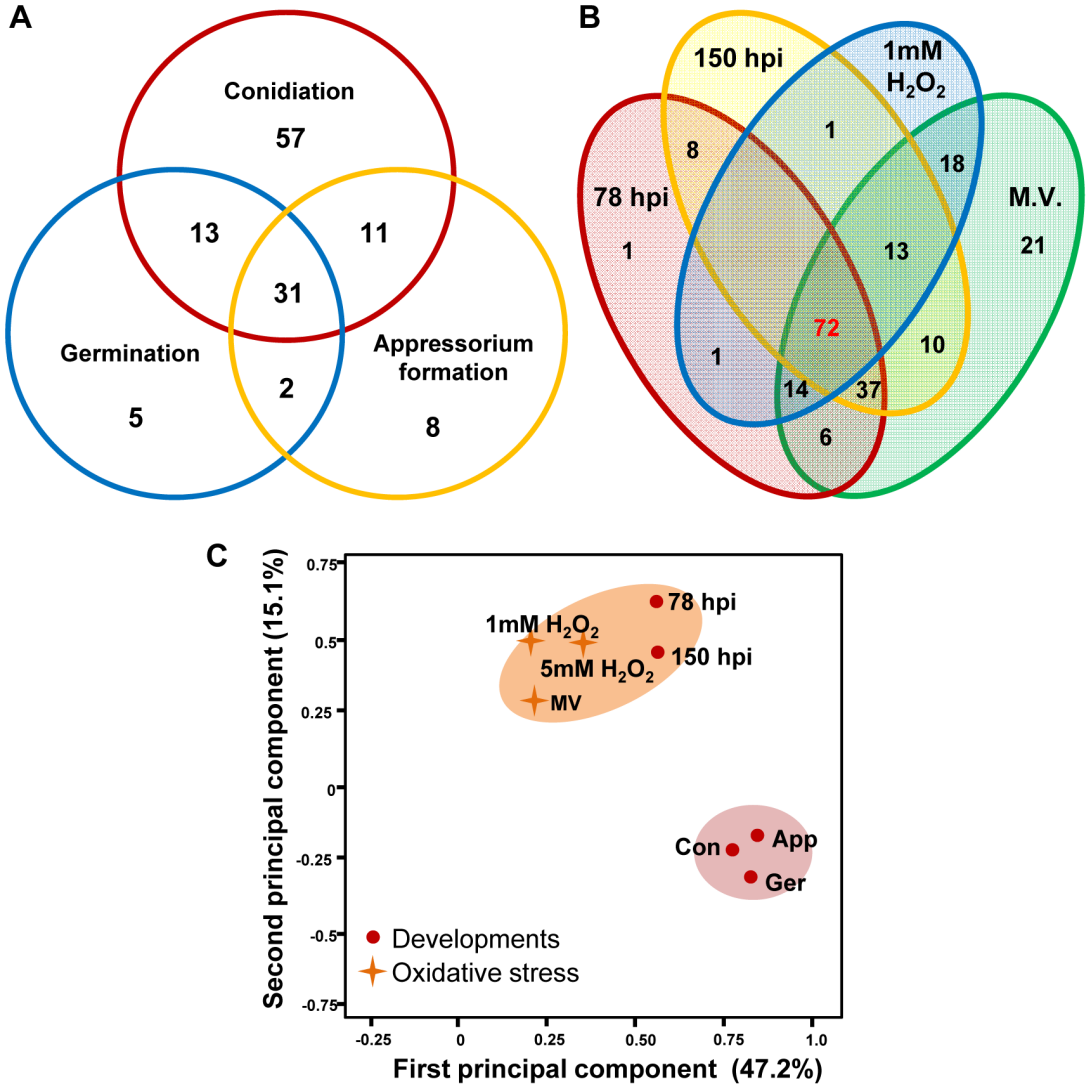
Venn diagrams showing up-regulated TF genes at infection-related developmental stages and under *in vitro* oxidative stress conditions. (A) The number of genes induced during conidiation (112), germination (51), and appressorium formation (52) is indicated. These TF genes are grouped into the following seven categories: (1) conidiation-associated up-regulation; (2) germination-associated up-regulation; (3) appressorium-associated up-regulation; (4) up-regulation during conidiation, germination, and appressorium formation; (5) up-regulation during conidiation and germination; (6) up-regulation during conidition and appressorium formation; (7) up-regulation during germination and appressorium formation. TF genes in groups 1 to 4 are listed in Table S5. (B) Venn diagram showing the number of genes up-regulated TF genes at 78 hpi and 150 hpi, and in response to two sources of oxidative stress, 1 mM H_2_O_2_ and methyl viologen. (C) PCA of the expression data from 206 TF genes in the following conditions: conidiation [Con], conidial germination [Ger], appressorium formation [App], infection (78 hpi and 150 hpi), 1 mM H_2_O_2_, 5 mM H_2_O_2_, and 10 mM methyl viologen [MV].

To identify TF genes that potentially control infection-related fungal development, we analyzed TF expression patterns during conidiation and/or in conidia, conidial germination, and appressorium formation. We identified 127 genes (61.7%) that were up-regulated during at least one of these developmental stages (Figure 3A). Expression of 70 genes was up-regulated at a single stage only: 57 (conidiation and/or in conidia), 5 (conidial germination), and 8 (appressorium formation). *MoHOX2*, a previously reported conidiation-specific TF gene [12], belonged to the first group. Thirty-one genes were found to be up-regulated at all three stages, and interestingly and included MGG_00021.6, a gene that is present exclusively in *M. oryzae* (Table S5).

### *In planta* proliferation and oxidative stress responses appear to share common regulatory machineries

To colonize host plants successfully, pathogens must overcome host-generated, defense-associated compounds such as reactive oxygen species (ROS) [42], [43]. To test the potential correlation between infectious growth *in planta* and oxidative stress responses, we compared the expression profiles under these conditions (Figure 3B). During infectious growth, 139 (67.5%) and 141 (68.4%) genes were up-regulated at 78 hpi and 150 hpi, respectively with 117 (71.8%) being up-regulated at both time points. Treatment with H_2_O_2_ or methyl viologen up-regulated 117 genes (71.8%), in which 61.5% of them (72) were also induced during *in planta* proliferation (Figure 3B). To further analyze this correlation, PCA was conducted with the data from five infection-related conditions and oxidative stresses caused by H_2_O_2_ and methyl viologen. The data from 78 hpi and 150 hpi and these oxidative stress conditions were separated from those collected during conidiation and/or in conidia, conidial germination, and appressorium formation (Figure 3C), further supporting a close relationship between infectious growth and oxidative stress responses.

**Figure 4:**
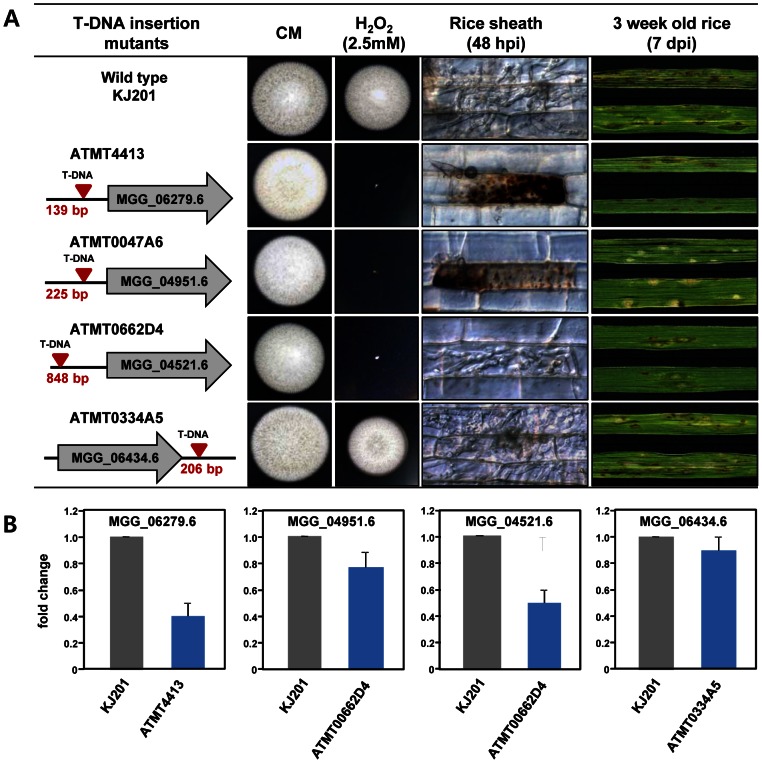
Functional analysis of selected TF genes. Phenotypes of T-DNA insertion mutants in four genes up-regulated at 72 hpi (MGG_06279.6, MGG_04951.6, MGG_04521.6, and MGG_06434.6). (A) The T-DNA insertion sites for each mutated gene, and mutant phenotypes, including sensitivity to H_2_O_2_, infectious growth in rice sheath and disease symptoms in 3 week old rice seedling, are shown. (B) Quantitative RT-PCR analysis of transcripts from the four TF genes in the corresponding mutant.

To validate the functional significance of these 72 genes during infectious growth and oxidative stress responses, we retrieved mutants in four genes, ATMT4413 (MGG_06279.6, Zn_2_Cys_6_ family), ATMT0047A6 (MGG_04951.6, Zn_2_Cys_6_ family), ATMT0662D4 (MGG_04521.6, GATA family), and ATMT0334A5 (MGG_06434.6, Myb family), from a *M. oryzae* T-DNA insertion mutant library [44]. Compared to wild-type strain, three of the mutants (ATMT4413, ATMT0047A6, and ATMT0662D4) with an insertion upstream of the open reading frame (ORF) showed increased sensitivity to 2.5 mM H_2_O_2_ (Figure 4A). These mutants also exhibited impaired infectious growth in rice, resulting in decreased virulence. However, one mutant (ATMT0334A5), with a T-DNA insertion at the 206 bp downstream from the stop codon of MGG_06434.6, was insensitive to 2.5 mM H_2_O_2_ and was nearly identical with wild-type strain KJ201 in terms of infectious growth and virulence (Figure 4A). Because all four mutants had a T-DNA insertion outside of ORF, we hypothesized that the phenotypes observed, except that of ATMT0334A5, were most likely caused by reduced expression of the tagged genes. To test this hypothesis, we examined their expression using qRT-PCR. The level of transcripts from the disrupted gene in the mutants in ATMT4413, ATMT0047A6, and ATMT0662D4 was reduced to 60%, 20% and 50%, respectively, of the corresponding wild-type level (Figure 4B). These results supported a strong correlation between expression profiles and function and suggested the involvement of largely overlapping sets of TFs in controlling pathogenicity and ROS stress responses.

### Functional analysis of conidiation-specific TF genes

**Figure 5:**
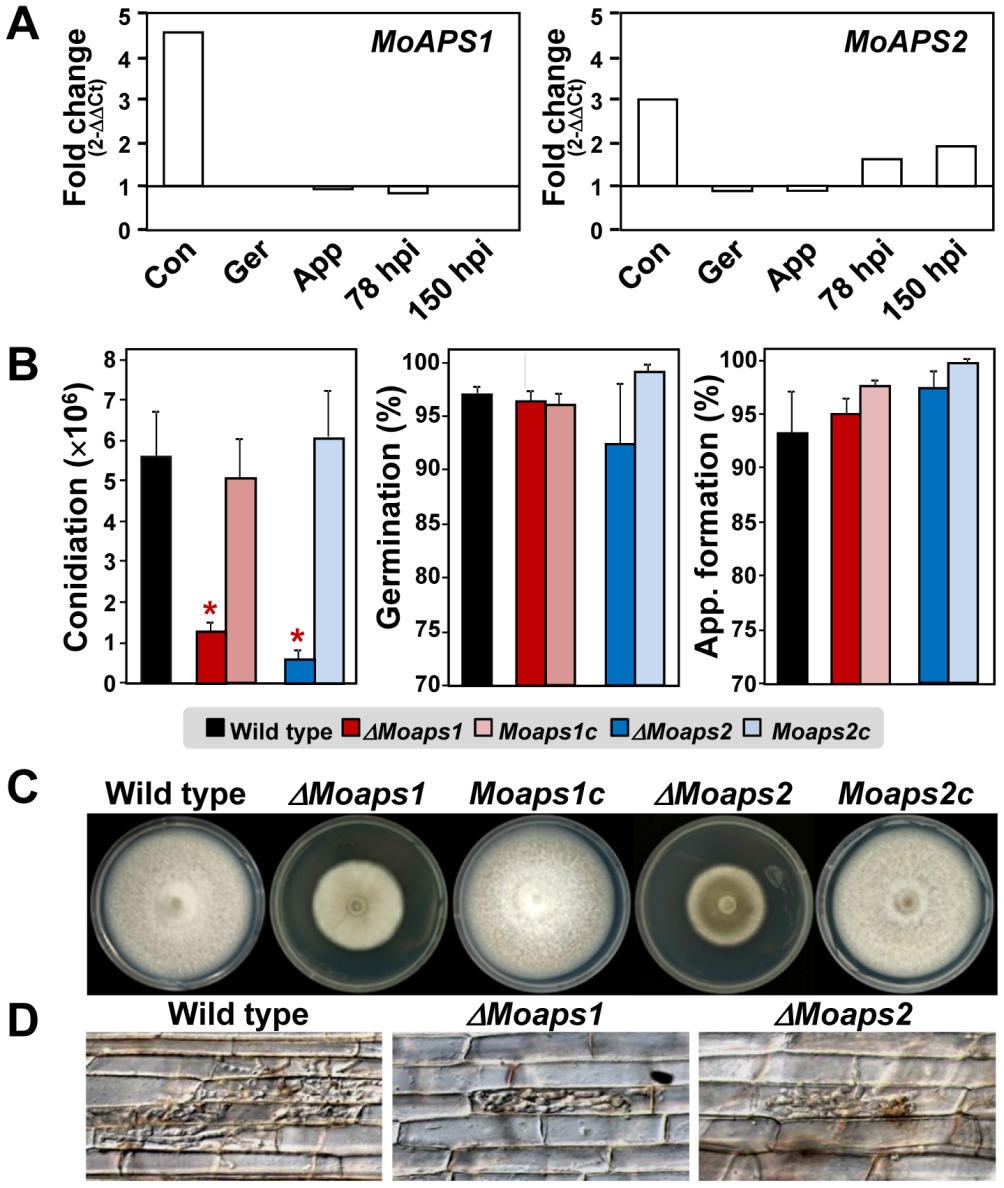
Phenotype analyses of *ΔMoaps1*, *ΔMoaps2*, and complemented mutants. (A) Expression patterns of *MoAPS1* and *MoAPS2* under five conditions: Con, conidiation; Ger, conidial germination; App, appressorium formation; and 78 hpi and 150 hpi. (B) Conidial production, conidial germination, and appressorium formation (left to right). The asterisk denotes a significant difference (at *P*<0.05). (C) Vegetative growth on CM agar. (D) Infectious growth in rice sheaths.

Two members of the fungal-specific APSES family, *MoAPS1* (MGG_09869.6) and *MoAPS2* (MGG_08463.6) (Figure S4) are up-regulated specifically during conidiation and/or in conidia (Figure 5A). Deletion of these genes (Figure S4C and S4E) caused a significant reduction in conidiation. In addition, the *ΔMoaps1* and *ΔMoaps2* mutants showed reduced vegetative growth (Figure 5C) and infectious growth in rice sheath cells (Figure 5D), resulting in 50% reduction in virulence. However, conidial germination and appressorium formation were normal (Figure 5B). All of the mutant phenotypes of *ΔMoaps1* and *ΔMoaps2* were restored by genetic complementation.

To further validate the utility of predicting functional roles based on expression profiles, we studied T-DNA insertion mutants of eight additional conidiation-specific TF genes (see Table S6). All eight mutants were defective in conidiation or conidial morphology with some additional defect in conidial germination, appressorium formation or pathogenicity (Figure S5). Conidiation of four mutants, ATMT0094A6 (MGG_06243.6, Zn_2_Cys_6_ family), ATMT0104A6 (MGG_02474.6, C_2_H_2_ family), ATMT0068B3 (MGG_01426.6, Myb family), and ATMT 0349D2 (MGG_02755.6, GATA family), was significantly reduced, and one previously reported mutant, (ATMT0651A4 (*MoHOX2*)) [12], did not produce any conidia. The remaining three mutants, ATMT0052B2 (MGG_06355.6, Zn_2_Cys_6_ family), ATMT0591D1 (MGG_09263.6, Zn_2_Cys_6_ family), and ATMT0034B1 (MGG_06507.6, C_2_H_2_ family), produced abnormally shaped conidia. Taken together, the phenotypes of both groups of mutants strongly support the value of expression patterns of TF genes in predicting their functions.

### Expression patterns of the 57 conidiation-specific genes during conidiogenesis

In *M. oryzae*, conidiogenesis is generally divided into four stages: (A) generation of conidiophores; (B) formation of a single-celled young conidium at the tip of conidiophore; (C) maturation of a three-celled conidium; and (D) multiplication of conidia in a sympodial manner [45]. To investigate expression patterns of these 57 genes at these stages, we collected samples at four different time points after induction of conidiation (Figure S6A). The time point at 0 h corresponded to submerged mycelial cultures in liquid CM which inhibits conidiogenesis [45], [46]. No conidia were observed at 6 h after induction of conidiation. Whereas, one to three-celled conidia were detected (5.3±3.1×10^4^ conidia/plate) at 12 h. After 18 h, many of typical three-celled conidia were detected (26.7±1.5×10^4^ conidia/plate). Finally, conidia were produced abundantly (756.7±20.5×10^4^ conidia/plate) at 24 h time point (Figure S6B). These observations were illustrated in Figure S6C.

To test whether these samples were suitable for stage-specific gene expression profiling during conidiogenesis, we examined expression patterns of three well known conidiogenesis-related genes, *COS1* [14], *CON7* [16], and *ACR1* [47]. Fold change in expression was calculated by dividing the expression level at 6 to 24 h by that at 0 h. Expression of all three genes increased during conidiation and/or in conidia. Increased *COS1* transcripts were first detected at 6 h. Levels of *Con7* and *ACR1* transcripts increased (≥2 fold) after 12 h. In particular, the amount of *ACR1* transcripts at 24 h was 17 times higher than that at 0 h (Figure S6D). These results are consistent with data in previous studies [14], [16], [47], supporting that our samples were suitable for detailed gene expression analyses during conidiogenesis.

All 57 conidiation-specific TF genes showed increased transcripts (≥2 fold) at more than one stage (Table S6). Seven genes (MGG_07319.6, MGG_00139.6, MGG_02447.6, MGG_07681.6, MGG_09263.6, MGG_01833.6, and MGG_06243.6) showed increased transcript levels at all four time points compared with that at 0 h, while 21 genes increased transcripts at only one of the time points (one gene at 6 h, one at 12 h, 10 at 18 h, and nine at 24 h). The rest of the genes had increased transcripts at two to three time points (three at 6 h, 12 at 18 h, 14 at 12 h, 18 h, and 24 h, nine at 12 and 18 h, one at 18 h and 24 h, and two at 12 h and 18 h). This data clearly showed differential expression of all 57 conidiation-specific TF genes conidiogenesis, suggesting their involvement in this process.

### Regulatory network of conidiation-specific TF genes

**Figure 6:**
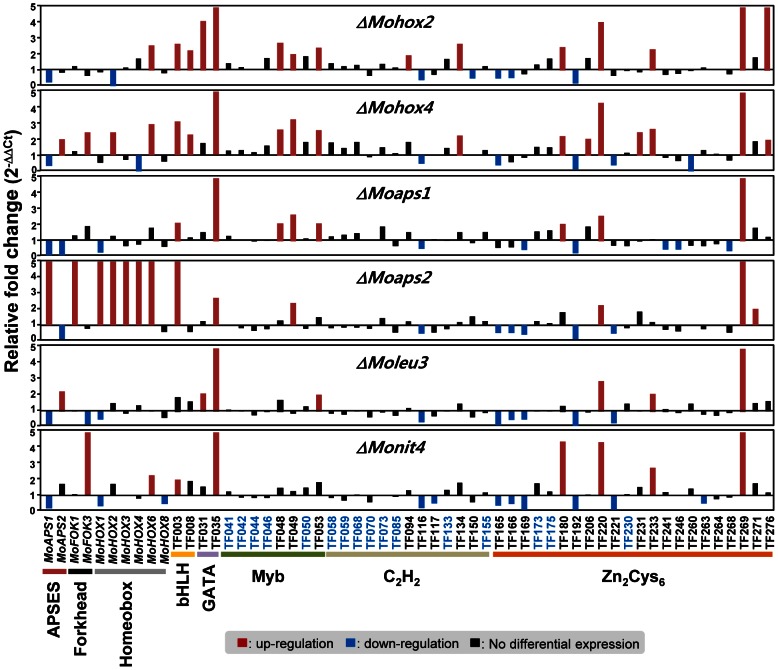
Expression profiles of 57 conidiation-specific TF genes in six TF gene deletion mutants. The mutants included *ΔMoaps1*, *ΔMoaps2*, *ΔMohox2*, *ΔMohox4*, *ΔMoleu3*, and *ΔMonit4*. Up-regulated genes in the mutants (more than 2 fold) are indicated by red bars, and down-regulated genes (less than 0.5 fold) are noted by blue bars. The genes that did not show differential expression in the six mutants are marked in blue.

To investigate the regulatory network controlling the expression and interactions of these 57 genes during conidiation and/or in conidia, we examined their expression in six TF gene deletion mutants. These mutants showed conidiation-related phenotypes such as no conidial production (*ΔMohox2* [12]), smaller conidia (*ΔMohox4* [12]), and reduced conidial production (*ΔMoaps1*(this study), *ΔMoaps2* (this study), *ΔMoleu3* [48], and *ΔMonit4* [48]). We compared gene expression profiles of these 57 genes in the six mutants with those in KJ201 to determine if and how their gene expression was affected by each mutation (Figure 6). Sixteen genes (Figure 6) were not affected by any of the mutations.

**Figure 7:**
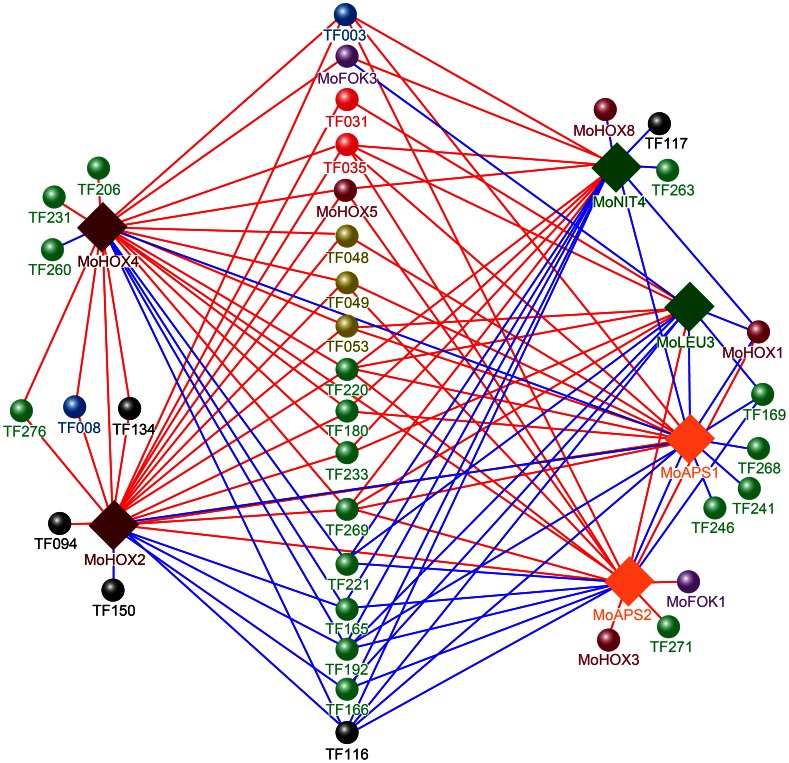
A model for the regulatory network controlling the expression of conidiation-specific TF genes. Solid diamonds indicate the genes deleted in *ΔMohox2*, *ΔMohox4*, *ΔMoaps1*, *ΔMoaps2*, *ΔMoleu3*, and *ΔMonit4*. Spheres correspond to up-regulated (red line) or down-regulated (blue line) TF genes in one or more of these mutants. Different colors of the sphere indicate different TF families: Green (Zn_2_Cys_6_); black (C_2_H_2_); violet (Homobox); orange (APSES); red (GATA); blue (bHLH); olive (Myb); violet (Forkhead). A detailed description of these genes is shown in Tables S5 and S6.

Among the remaining 41 genes, TF116 (MGG_02474.6, C_2_H_2_ family) and TF192 (MGG_03711.6, Zn_2_Cys_6_) were down-regulated in all mutants, suggesting that their expression requires the mutated genes, whereas three genes, including TF035 (MGG_07319.6, GATA type), TF220 (MGG_06243.6, Zn_2_Cys_6_), and TF269 (MGG_09829.6, Zn_2_Cys_6_), were up-regulated in all mutants. Expression of several genes were up- or down-regulated only in one mutant: TF094 (MGG_00373.6, C_2_H_2_) and TF150 (MGG_06507.6, C_2_H_2_) in Δ*Mohox2*; TF206 (MGG_04951.6, Zn_2_Cys_6_), TF260 (MGG_09263.6, Zn_2_Cys_6_), TF231 (MGG_07131.6, Zn_2_Cys_6_) in Δ *Mohox4*; TF241 (MGG_07681.6, Zn_2_Cys_6_), TF246 (MGG_08094.6, Zn_2_Cys_6_), and TF268 (MGG_09825.6, Zn_2_Cys_6_) in Δ*Moaps1* ;TF271 (MGG_09950.6, Zn_2_Cys_6_), *MoFOK1*, *MoHOX3 in* Δ*MoAPS2*; TF263 (MGG_09312.6, Zn_2_Cys_6_), TF117 (MGG_02505.6, C_2_H_2_), and *MoHOX8* in Δ*Monit4*. In addition, expression of TF134 (MGG_02845.6, C_2_H_2_), TF008 (MGG_10837.6, bHLH), and TF276 (MGG_10528.6, Zn_2_Cys_6_) seems to require both *MoHOX2* and *MoHOX4*, while *MoHOX1* requires only *MoAPS2* and is down-regulated in *ΔMoaps1*, *ΔMoleu3* and *ΔMonit4*. Based on the results shown in Figure 6, we developed a model for the regulatory network controlling the expression of conidiation-specific TF genes (Figure 7).

## Discussion

Advances in tools for analyzing global gene expression profiles have facilitated the identification of genes potentially associated with specific processes and the characterization of regulatory networks controlling their expression. To test whether expression patterns of TF genes under diverse conditions help predict the functional roles of individual genes and potential regulatory interactions among them, we analyzed expression of 206 *M. oryzae* TF genes under 32 conditions using qRT-PCR. Expression profiles and functional validation of several genes selected based on their expression patterns clearly demonstrate the value of TF gene expression patterns in predicting their function. This comprehensive expression data of TF genes, publicly available through FTFD, will serve as a new community resource in analyzing the functions of and potential interactions among individual TF genes.

Previous studies based on microarrays [49], [50], SAGE [51], or RNA-seq [52] revealed many genes that potentially play important roles under specific conditions in *M. oryzae*. However, despite the biological significance of TF genes, relatively few have been characterized in *M. oryzae* and their regulation and genetic interactions have not been systematically investigated. In this study, we adopted qRT-PCR to address this deficiency. This method is labor intensive but has been shown to be robust in accurately quantifying TF transcripts [4]. We have identified differentially expressed TF genes under 32 conditions with most of them being up-regulated under at least one of these conditions (Figure 2).

Conidiation in plant pathogenic fungi, including *M. oryzae*, plays a central role in their life and disease cycles and epidemics. However, little is known about the molecular changes underpinning conidiation in *M. oryzae*. The developmental complexity of conidiation was suggested by the fact that 8.5% of the protein-coding genes in *M. oryzae* are differentially expressed during conidiation and/or in conidia based on a whole-genome microarray experiment [46]. Approximately 25% of the predicted genes are differentially expressed during conidiation in *Neurospora crassa* [53] and that ∼1,000 genes in *Aspergillus nidulans* are involved in conidiation [54]. Thus, it is likely that a relatively large numbers of TF genes are involved in controlling and coordinating the expression of many genes that participate in producing conidia. Our analysis revealed that more TF genes were up-regulated during conidiation and/or in conidia (112 genes) than during conidial germination (51 genes) and appressorium formation (52 genes). However, most of the genes induced during conidial germination and appressorium formation were also induced during conidiation and/or in conidia, suggesting that the same general transcription regulators probably control multiple developmental changes.

In total, 57 genes were considered conidiation-specific. These 57 genes were differentially expressed at one or more stages of conidiation, including conidiophore formation, conidia formation, and multiplication of conidia in a sympodial manner (Figure S6). The importance of many of these genes (41 out of 57) in conidiation was implied by their modified expression in one or more mutants that are defective in conidiation. Compared with the patterns observed in the wild-type strain KJ201, three genes were up-regulated while two genes were down-regulated in all the mutants during conidia production and/or in conidia. We hypothesize that these TFs act as major regulators of transcription throughout conidiation. These genes are interesting candidates for functional studies via mutagenesis. Results from this gene expression analysis in the multiple mutant backgrounds led to a model for a regulatory network controlling the expression of conidiation-specific TF (Figure 7). This model will serve as a useful roadmap in studying the regulation of conidiation.

Interestingly, most of the TF genes induced by oxidative stresses were also induced during *in planta* growth (72 genes, Figure 3B); this finding is consistent with the accumulating evidence suggesting that fungal pathogens must overcome plant-generated ROS for successful invasion [20], [26], [42], [55]. Our results also indicate that *in vitro* oxidative stress conditions mimic those that the fungus encounters *in planta*, and that *in planta* invasion and *in vitro* oxidative stress responses share common transcriptional regulatory factors.

Nitrogen starvation is known to be one of the important environmental cues for appressorium formation and *in planta* growth of *M. oryzae* [50]. Donofrio et al [50] reported that one GATA family TF gene, *NUT1* (MGG_06050.6), was highly up-regulated in both nitrogen starvation condition and inside infected rice, suggesting NUT1 is a global nitrogen regulator. We also found that 13 TF genes were up-regulated in response to nitrogen starvation as well as during host infection (data not shown). Moreover, one of the *M. oryzae* specific TF gene (MGG_00021.6, Zn2Cys6) and one Myb family TF gene (MGG_06898.6) showed up-regulation at all three developmental stages, two infection stages, and nitrogen starvation, suggesting that these TF genes function as general regulators controlling multiple processes in *M. oryzae*.

One of the most important outcomes of this study is demonstrating the value of expression data in predicting the putative function of individual TF genes. Those TF genes induced during conidiation and/or in conidia were used to test their value. *MoHOX2*, which plays a critical role in conidial production [12], was identified as a conidiation-specific TF gene. Further, T-DNA insertional mutants in seven of these genes were defective in conidiogenesis. Targeted mutagenesis of two fungal-specific TF genes of the APSES family, which are up-regulated during conidiation and/or in conidia, also caused defects in conidiation. In a second test involving four mutants in the TF genes induced both during infection and under oxidative stress also showed that the mutants displayed increased sensitivity to oxidative stress and severely reduced infectious growth in rice (Figure 4A). Results from both tests strongly supported the predictive value of expression patterns in functional studies.

Considering that similar TF expression profiles were observed between *in planta* infectious growth and oxidative stress, a high throughput *in vitro* assay system that screens for mutants defective in growth under oxidative stress can serve as a surrogate platform for quickly identifying candidate pathogenicity genes. Metal ions, such as MnCl_2_ and FeSO_4_, induced expression of many TF genes. The effect of metal ions in fungal biology and pathogenicity is not clearly understood. However, a recent study suggested that ferrous ion is required for the normal function of the *DES1* gene in *M. oryzae* [26]. In mammals, manganese ion induces apoptosis by causing endoplasmic reticulum stress and mitochondrial dysfunction [56], [57]. Comprehensive expression profiles of TF genes in the presence of metal ions or other abiotic stresses will help decipher not only how fungal responses to such stresses are controlled at the transcriptional level, but also their roles in fungal biology and pathogenicity.

Functional characterization of fungal genes requires a well-standardized platform that assays diverse phenotypes. However, only a few phenotypes, such as mycelial growth, reproduction, and pathogenicity, have been evaluated in gene functional studies with filamentous fungi [44], [58], [59]. When mutants of *N. crassa* in 103 TF genes were evaluated, only less than half of the resulting mutants exhibited clear phenotypes [59], which can be attributed to overlapped functions among TFs, limited phenotype assays, or a combination of both. In clusion of 26 abiotic stress conditions to profile expression patterns has helped the establishment of a novel phenomics platform for large-scale gene functional studies in *M. oryzae* and other pathogenic fungi. This platform will help systematically decipher the functional roles of TF genes in fungal development, pathogenicity, and abiotic stress management.

## Materials and Methods

### Identification of TF genes

Annotated genomes of 21 fungal and 2 Oomycete species (Table S1) were used to compare of the number and types of TF genes. Putative TF genes in version 6 of the *M. oryzae* genome (http://www.broadinstitute.org/annotation/fungi/magnaporthe) were identified using the annotation pipeline in FTFD which annotates fungal TFs based on the InterPro database using DNA binding motifs [22]. To identify *M. oryzae* specific TF genes (orphan genes), a combination of BLAST matrix [60] and InParanoid algorism [23] was used. We applied a cutoff e-value of less than 10^−50^ for protein similarity for BLAST matrix searches and the default parameter for InParanoid.

### Fungal isolates and developmental and stress conditions

*M. oryzae* KJ201 (wild-type strain) and all mutants used in this study were obtained from the Center for Fungal Genetic Resource (CFGR) at Seoul National University, Seoul, Korea. All strains were grown at 25°C for 14 days on oatmeal agar. Conidia and germinated conidia were harvested as described previously [61], and appressoria were collected 6 h after dropping a conidial suspension (5×10^4^ conidia/ml) on a hydrophobic surface. For infected plant samples, after inoculating rice seedlings (3–4 leaf stage) with 20 ml of a KJ201 conidial suspension (1×10^5^ conidia/ml), leaves were collected at 78 hpi and 150 hpi.

Prior to exposing fungal cultures to various types of stress, cultures of 100 ml liquid CM (complete medium) inoculated with 1 ml of a conidial suspension (5×10^4^ conidia/ml) were incubated at 25°C for 4 days in an orbital shaker (120 rpm). The resulting mycelia were harvested using a 0.45-µm filter, washed with sterilized distilled water, transferred to fresh liquid CM and minimal medium (MM) [62] as a control, and CM or MM containing each treatment (Table S4) for 4 h culture. All mycelial samples were harvested from three replicates of three biological repeats, immediately frozen using liquid nitrogen, and stored at −80°C until processed.

For harvesting samples at different time points during conidiogenesis, a previously described procedure [46] was slightly modified. Actively growing wild-type mycelia were inoculated into liquid CM, and incubated at 25°C on a 120 rpm orbital shaker for 4 days. The resulting mycelia were fragmented using spatula and pressed through two-layers of cheese cloth. The mycelia were collected using two-layers of miracloth (Calbiochem, California, USA) and washed three times with one liter of sterilized distilled water. After resuspending the harvested mycelia in 10 ml sterilized distilled water, 400 µl of the suspension was spread on each 0.45 µm pore cellulose nitrate membrane filter (Whatman, Maidstone, England) placed on V8-Juice agar plate. The plates were incubated at 25°C with constant light. The whole tissue on the membrane filters was collected at 0 h, 6 h, 12 h, 18 h, and 24 h after inoculation by disposable scraper (iNtRON Biotechnology, Seoul, Korea). All samples were harvested from three replicates of three biological repeats, immediately frozen using liquid nitrogen, and stored at −80°C until processing.

### Nucleic acid manipulation and qRT-PCR

Total RNA was extracted using an Easy-Spin Total RNA Extraction Kit (iNtRON Biotechnology, Seoul, Korea), and 5 µg of RNA was reverse-transcribed to cDNA using the Prom-II Reverse Transcription System (Promega, Madison, WI, USA) according to the manufacturer's instructions. The resulting cDNA preparations were diluted to 12.5 ng/µl and kept at −20°C. A total of 206 primer pairs were designed using the 3′-end exon region of the target genes (GC contents = 45–55% and Tm = 60) (Table S7).

qRT-PCR reactions were performed using a MicroAmp Optical 96-Well Reaction Plate (PE Biosystems, Foster City, CA, USA) and an Applied Biosystems 7500 Real-Time PCR System. Each well contained 5 µl of Power 2× SYBR Green PCR Master Mix (Applied Biosystems, Warrington, UK), 2 µl of cDNA (12.5 ng/µl), and 15 pmol of each primer. The thermal cycling conditions were 10 min at 94°C followed by 40 cycles of 15 s at 94°C and 1 min at 60°C. All amplification curves were analyzed with a normalized reporter threshold of 0.1 to obtain the threshold cycle (Ct) values.

### Data analyses

To identify an appropriate reference gene for normalizing the expression levels of individual TF genes, GeNorm v.3.4 [33], Normfinder [34] and BestKeeper [35] were used. Expression levels of the chosen reference gene, *β-tubulin*, were measured in more than two replicates for each PCR run, and their average Ct value was used for relative expression analyses.

To compare the relative abundance of target gene transcripts, the average Ct value was normalized to that of *ß-tubulin* for each of the samples as 2^−ΔCt^, where −ΔCt = (Ct of the target gene – Ct of *ß-tubulin*). Fold changes of transcripts in samples representing developmental stages and infectious growth relative to those in mycelial samples in liquid CM were calculated as 2^−ΔΔCt^, where −ΔΔCt = (Ct of the target gene –Ct of *ß-tubulin*) test condition - (Ct of the target gene – Ct of *ß-tubulin*) CM [41]. qRT-PCR was conducted twice with three replicates, and all data are presented. The fold changes of transcripts from various stress-exposed mycelial samples compared to those in untreated samples (CM or MM) were calculated as 2^−ΔΔCt^, where −ΔΔCt = (Ct of the target gene –Ct of *ß-tubulin*) treated condition - (Ct of the target gene – Ct of *ß-tubulin*) untreated condition.

Pearson's correlation coefficient and Spearman's rank were used to measure the similarity between gene expression profiles and the similarity between samples, respectively. A heat map of the clustered genes and samples was generated by complete linkage. A principle component analysis (PCA) was conducted to reduce the dimensions and to understand the relationships between the TF genes and the experimental conditions. PCA was performed using SPSS software v.12.0 (SPSS Inc., Chicago, IL, USA).

To build a model for the regulatory network controlling the expression of conidiation-specific TF genes based on their expression patterns in six TF gene deletion mutants, we used NodeXL (http://nodexl.codeplex.com).

### *In vitro* growth assay, monitoring of infectious growth, and pathogenicity assays

Assays for measuring the sensitivity to exogenous oxidative stress were performed on CM agar amended with 2.5–5 mM H_2_O_2_ or methyl viologen. Radial colony growth was measured on day 6 after inoculation. Infection assays with rice sheath and 3-week-old rice seedlings were conducted as described previously [63].

### Generation and characterization of deletion mutants in two TF genes

Gene disruption (Fig. S4B and D) and fungal transformation were conducted as described previously [61]. Putative mutants were confirmed by Southern blot analysis. Vegetative growth, pigmentation, conidiation, conidial size, conidial germination, appressorium formation, and infection assays on onion epidermis, rice sheath cells, and rice seedlings were conducted as described previously [12], [63].

## Supporting Information

Figure S1 **Evaluation of candidate reference genes for RT-PCR analyses.** The gene codes and primers used for the qRT-PCR are given in Table S3. Transcripts from seven genes were measured under 32 conditions (Table S4). (A) Data from box plot analysis are shown. (B) Absolute Ct values under 32 conditions are shown.
(PDF)

Figure S2 **Expression stability of candidate reference genes calculated using the GeNORM program.** Expression data were used to calculate average expression stability (M) values. A lower M value indicates more stable expression. (A) The X axis indicates the rank of these genes according to their expression stability under three developmental stages, two infected stages, oxidative stress, nutrient utilization, ionic stresses, ambient pH, DNA repair, phenolic compound, others and the overall rank. (B) A graphical presentation of the M values.
(PDF)

Figure S3 **Expression patterns of the** ***MPG1*** **and** ***DES1*** **genes in wild-type strain KJ201 under 31 different conditions.** Fold changes of (A) *MPG1* and (B) *DES1* under these conditions are presented. Experimental conditions and abbreviations are listed in Table S4.
(PDF)

Figure S4 **Phylogenetic positions of two APSES TF genes (** ***MoAPS1*** **and** ***MoAPS2*** **) and mutagenesis strategy.** (A) A neighbor-joining tree was constructed based on the amino acid sequences of representative fungal APSES TFs. The numbers at the nodes indicate bootstrap values (%) in 10,000 bootstrap replicates. Clades containing *MoAPS1*, *MoAPS2* and *MstuA*, respectively are differently shaded. Red and orange boxes denote the DNA binding domain (IPR003163) and Ankyrin repeat domain (IPR002210), respectively. The abbreviations for the fungal species included in this analysis (followed by GenBank accession numbers) are: Mo, *M. oryzae*; Pm, *Penicillium marneffeip*; An, *Aspergillus nidulans*; Wd, *Wangiella dermatitidis*; Nc, *Neurospora crassa*; Fo, *Fusarium oxysporum*; Ca, *Candida albicans*; Sc, *Saccharomyces cerevisiae*; Sp, *Schizosaccharomyces pombe*. The *MoAPS1* (MGG_009869.6; B) and *MoAPS2* (MGG_008363.6; D) gene were replaced with the *hph* cassette via homologous recombination. (C) To confirm the disruption of *MoAPS1*, genomic DNA samples were digested with *Eco*RI and probed with a fragment corresponding the 3′ flanking region (marked by a red bar). (E) For *MoAPS2*, genomic DNA samples were digested with *Sal*I and hybridized with a probe at the 5′flanking region.
(PDF)

Figure S5 **T-DNA insertion sites in eight mutants defective in conidiation-specific TF genes and resulting phenotypes.** Phenotypes of these mutants are derived from *Magnaporthe oryzae* T-DNA insertion mutant library (http://atmt.snu.ac.kr).
(PDF)

Figure S6 **Conidiation at four different time points.** (A) Pictures of fungal culture on cellulose nitrate membrane filter laid with on V8-Juice agar medium from 0 h to 24 h. (B) Numbers of conidia (per plate) at the indicated time. (C) Diagram illustrating conidiation process based on microscopic observation. Three genes involved at different stages of conidition, which are based on a study by Liu *et al.* (2010), are noted. (D) Expression patterns of these three genes during conidiation and/or in conidia in wild-type KJ201 are shown.
(PDF)

Table S1 **Transcription factors encoded by 23 fungal and Oomycete genomes.**
(PDF)

Table S2 **Distribution of putative homologues of 26** ***Magnaporthe oryzae*** **-specific transcription factor genes analyzed using Blast Matrix and InParanoid algorism.**
(PDF)

Table S3 **Primers used to amplify potential reference genes needed for gene expression analyses.**
(PDF)

Table S4 **Conditions used to extract RNA samples for gene expression analyses.**
(PDF)

Table S5 **List of TF genes up-regulated under developmental conditions.**
(PDF)

Table S6 **Relative abundance of transcripts from the 57 conidiation-specific TF genes during conidiation and/or in conidia.**
(PDF)

Table S7 **Primers used for quantitative real-time PCR.**
(PDF)
