# Supplementary material for: Global Expression Profiling of Transcription Factor Genes Provides New Insights into Pathogenicity and Stress Responses in the Rice Blast Fungus
Source: PLoS Pathog. 2013 Jun 6;9(6):e1003350. doi: 10.1371/journal.ppat.1003350 (PMC3675110; doi:10.1371/journal.ppat.1003350)
Supplement: Figure S1 — Evaluation of candidate reference genes for RT-PCR analyses. The gene codes and primers used for the qRT-PCR are given in Table S3. Transcripts from seven genes were measured under 32 conditions (Table S4). (A) Data from box plot analysis are shown. (B) Absolute Ct values under 32 conditions are shown. (PDF) [file ppat.1003350.s001.pdf]

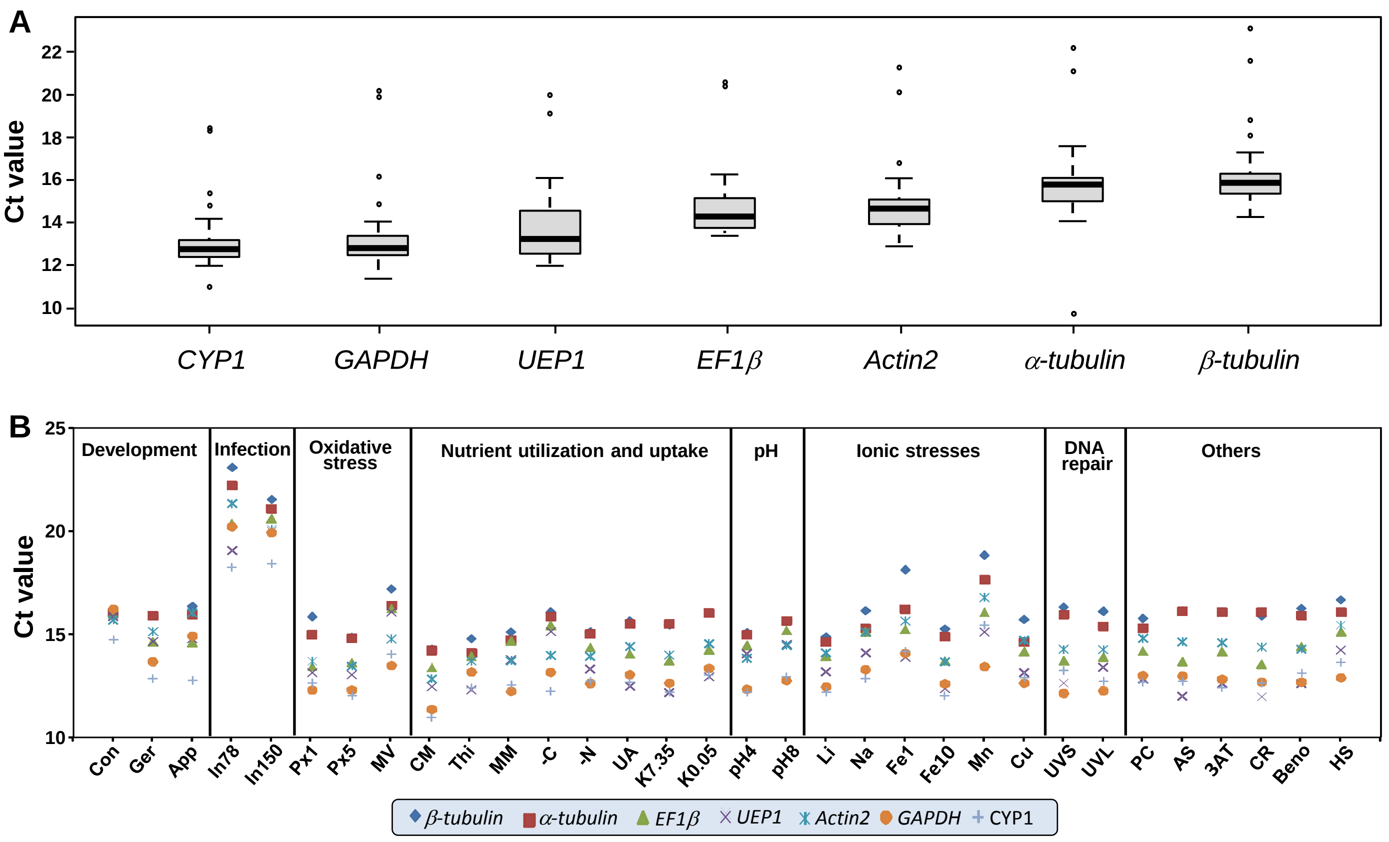

Figure S1. Evaluation of candidate reference genes for RT-PCR analyses. The gene codes and primers used for the qRT-PCR are given in Table S3. Transcripts from seven genes were measured under 32 conditions (Table S4). (A) Data from box plot analysis are shown. (B) Absolute Ct values under 32 conditions are shown.
