# Supplementary material for: Global Expression Profiling of Transcription Factor Genes Provides New Insights into Pathogenicity and Stress Responses in the Rice Blast Fungus
Source: PLoS Pathog. 2013 Jun 6;9(6):e1003350. doi: 10.1371/journal.ppat.1003350 (PMC3675110; doi:10.1371/journal.ppat.1003350)
Supplement: Figure S2 — Expression stability of candidate reference genes calculated using the GeNORM program. Expression data were used to calculate average expression stability (M) values. A lower M value indicates more stable expression. (A) The X axis indicates the rank of these genes according to their expression stability under three developmental stages, two infected stages, oxidative stress, nutrient utilization, ionic stresses, ambient pH, DNA repair, phenolic compound, others and the overall rank. (B) A graphical presentation of the M values. (PDF) [file ppat.1003350.s002.pdf]

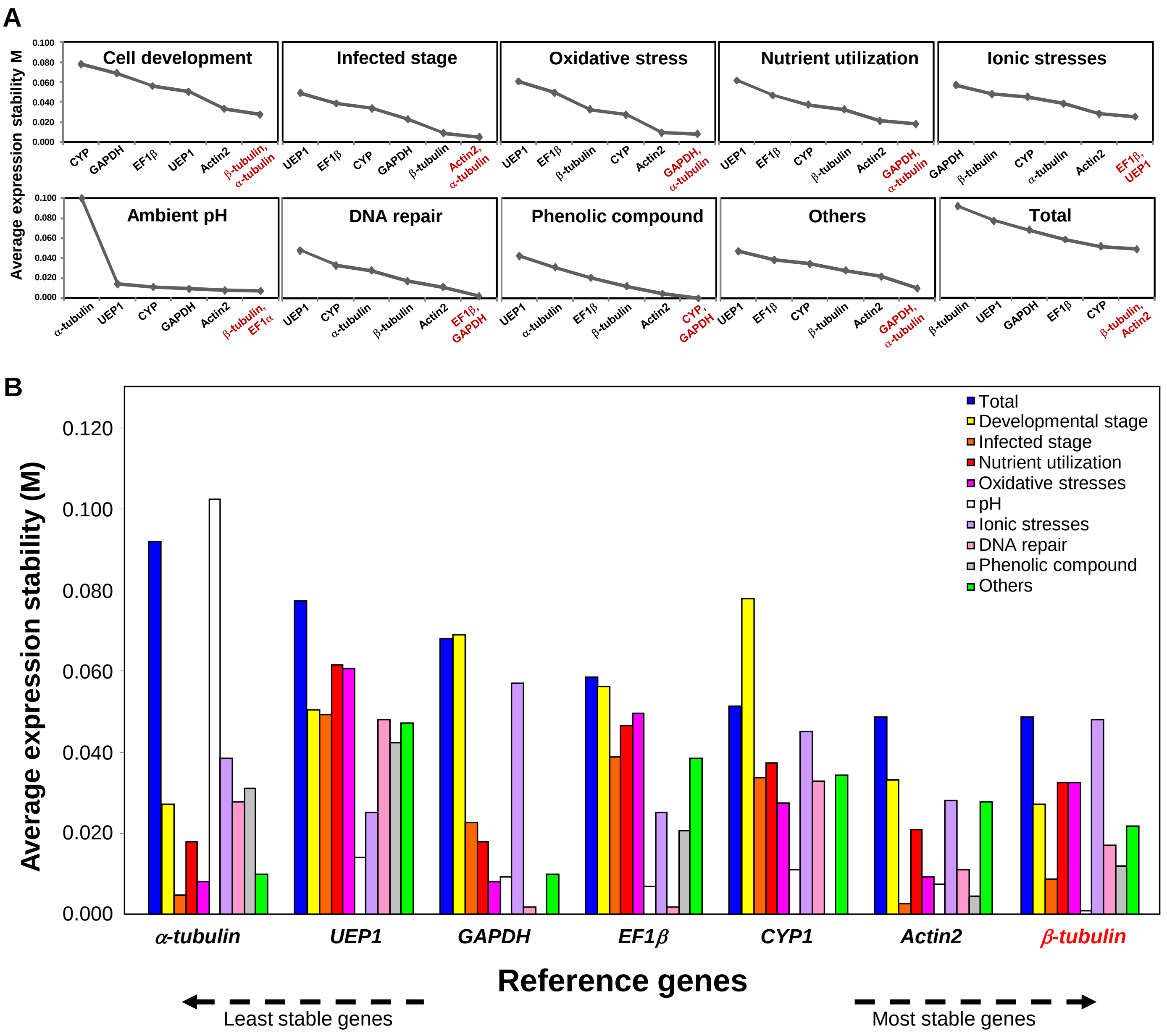

Figure S2. Expression stability of candidate reference genes calculated using the GeNORM program. Expression data were used to calculate average expression stability (M) values. A lower M value indicates more stable expression. (A) The X axis indicates the rank of these genes according to their expression stability under three developmental stages, two infected stages, oxidative stress, nutrient utilization, ionic stresses, ambient pH, DNA repair, phenolic compound, others and the overall rak. (B) A graphical presentation of the M values.
