# Supplementary material for: Global Expression Profiling of Transcription Factor Genes Provides New Insights into Pathogenicity and Stress Responses in the Rice Blast Fungus
Source: PLoS Pathog. 2013 Jun 6;9(6):e1003350. doi: 10.1371/journal.ppat.1003350 (PMC3675110; doi:10.1371/journal.ppat.1003350)
Supplement: Figure S3 — Expression patterns of the MPG1 and DES1 genes in wild-type strain KJ201 under 31 different conditions. Fold changes of (A) MPG1 and (B) DES1 under these conditions are presented. Experimental conditions and abbreviations are listed in Table S4. (PDF) [file ppat.1003350.s003.pdf]

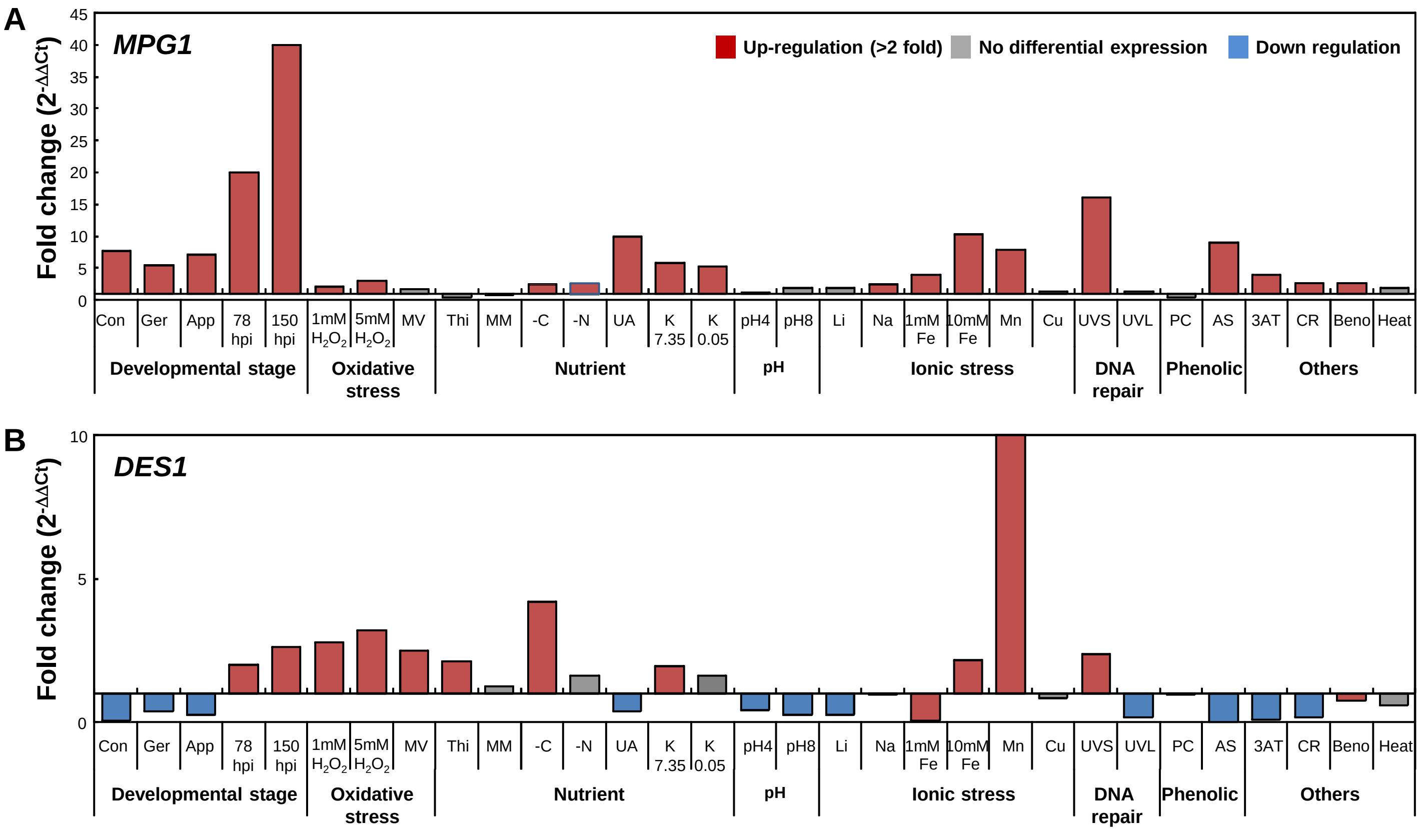


**Figure S3**. Expression patterns of the *MPG1* and *DES1* genes in wild type strain KJ201 under 31 different conditions. Fold changes of (A) *MPG1* and (B) *DES1* under these conditions are presented. Experimental conditions and abbreviations are listed in Table S4.
