# Supplementary material for: Global Expression Profiling of Transcription Factor Genes Provides New Insights into Pathogenicity and Stress Responses in the Rice Blast Fungus
Source: PLoS Pathog. 2013 Jun 6;9(6):e1003350. doi: 10.1371/journal.ppat.1003350 (PMC3675110; doi:10.1371/journal.ppat.1003350)
Supplement: Figure S4 — Phylogenetic positions of two APSES TF genes ( MoAPS1 and MoAPS2 ) and mutagenesis strategy. (A) A neighbor-joining tree was constructed based on the amino acid sequences of representative fungal APSES TFs. The numbers at the nodes indicate bootstrap values (%) in 10,000 bootstrap replicates. Clades containing MoAPS1, MoAPS2 and MstuA, respectively are differently shaded. Red and orange boxes denote the DNA binding domain (IPR003163) and Ankyrin repeat domain (IPR002210), respectively. The abbreviations for the fungal species included in this analysis (followed by GenBank accession numbers) are: Mo, M. oryzae; Pm, Penicillium marneffeip; An, Aspergillus nidulans; Wd, Wangiella dermatitidis; Nc, Neurospora crassa; Fo, Fusarium oxysporum; Ca, Candida albicans; Sc, Saccharomyces cerevisiae; Sp, Schizosaccharomyces pombe. The MoAPS1 (MGG_009869.6; B) and MoAPS2 (MGG_008363.6; D) gene were replaced with the hph cassette via homologous recombination. (C) To confirm the disruption of MoAPS1, genomic DNA samples were digested with EcoRI and probed with a fragment corresponding the 3′ flanking region (marked by a red bar). (E) For MoAPS2, genomic DNA samples were digested with SalI and hybridized with a probe at the 5′flanking region. (PDF) [file ppat.1003350.s004.pdf]

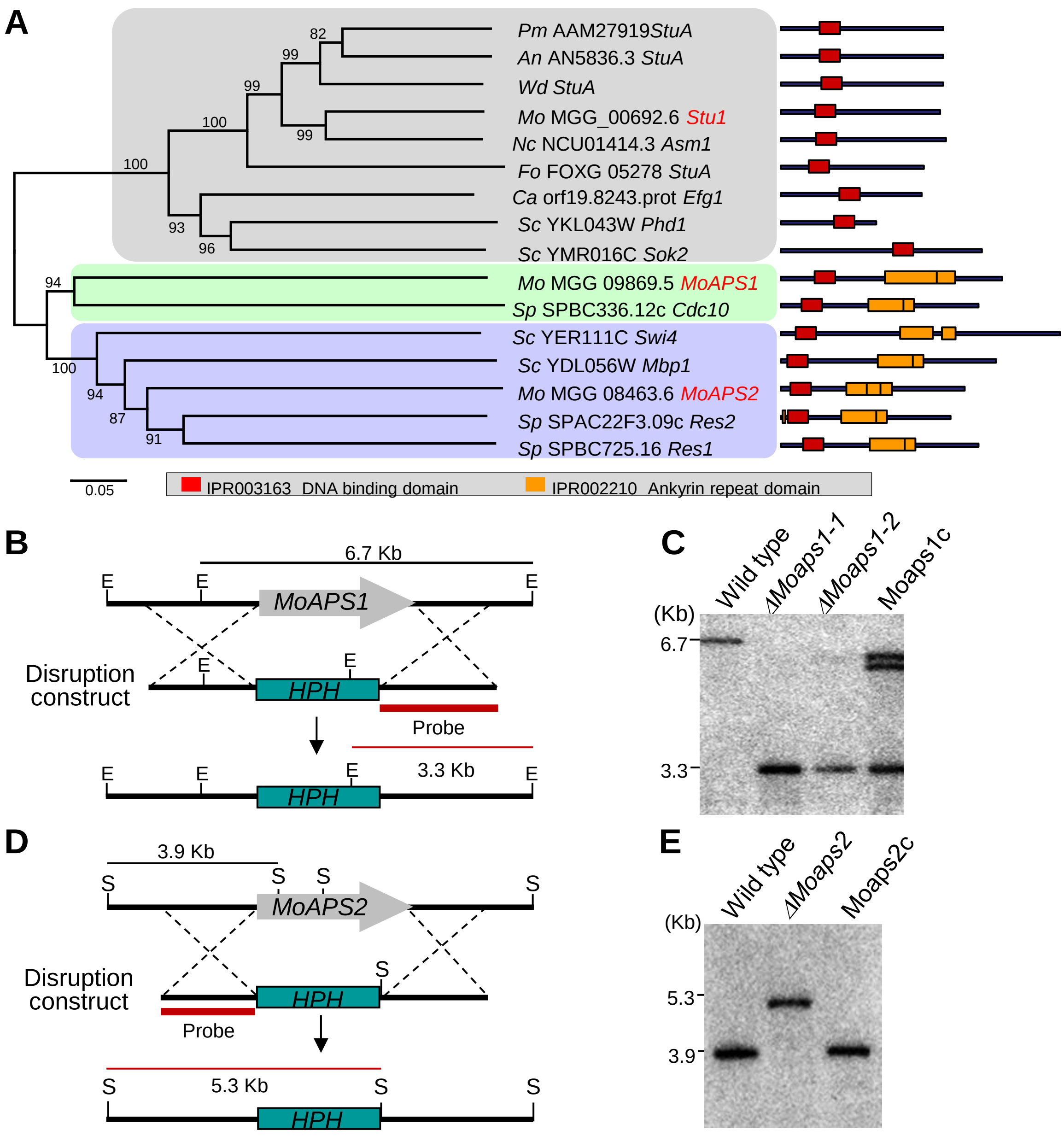

Figure S4. Phylogenetic positions of two APSES TF genes (*MoAPS1* and *MoAPS2*) and mutagenesis strategy. (A) A neighbor-joining tree was constructed based on the amino acid sequences of representative fungal APSES TFs. The numbers at the nodes indicate bootstrap values (%) in 10,000 bootstrap replicates. Clades containing *MoAPS1*, *MoAPS2* and *MstuA*, respectively are differently shaded. Red and orange boxes denote the DNA binding domain (IPR003163) and Ankyrin repeat domain (IPR002210), respectively. The abbreviations for the fungal species included in this analysis (followed by GenBank accession numbers) are: Mo, *M. oryzae*; Pm, *Penicillium marneffeip*; An, *Aspergillus nidulans*; Wd, *Wangiella dermatitidis*; Nc, *Neurospora crassa*; Fo, *Fusarium oxysporum*; Ca, *Candida albicans*; Sc, *Saccharomyces cerevisiae*; Sp, *Schizosaccharomyces pombe*. The *MoAPS1* (MGG_009869.6; B) and *MoAPS2* (MGG_008363.6; D) gene were replaced with the *hph* cassette via homologous recombination. (C) To confirm the disruption of *MoAPS1*, genomic DNA samples were digested with *Eco*RI and probed with a fragment corresponding the 3' flanking region (marked by a red bar). (E) For *MoAPS2*, genomic DNA samples were digested with *Sal*I and hybridized with a probe at the 5'flanking region.
