# Supplementary material for: Global Expression Profiling of Transcription Factor Genes Provides New Insights into Pathogenicity and Stress Responses in the Rice Blast Fungus
Source: PLoS Pathog. 2013 Jun 6;9(6):e1003350. doi: 10.1371/journal.ppat.1003350 (PMC3675110; doi:10.1371/journal.ppat.1003350)
Supplement: Figure S5 — T-DNA insertion sites in eight mutants defective in conidiation-specific TF genes and resulting phenotypes. Phenotypes of these mutants are derived from Magnaporthe oryzae T-DNA insertion mutant library (http://atmt.snu.ac.kr). (PDF) [file ppat.1003350.s005.pdf]

T-DNA insertion mutants	No. TFs	Conidiation	Conidial morphology	Conidial germination	Appressorium Formation	Pathogenicity
ATMT0651A4<br>T-DNA 673 bp MGG_00184.6	HOX2	No conidia	-	-	-	-
ATMT0094A6<br>T-DNA 511 bp MGG_06243.6	TF220	Reduced	Normal	<50%	<50%	No disease
ATMT0104A6<br>T-DNA 1350 bp MGG_02474.6	TF116	Reduced	Normal	>50%	No App.	Reduced
ATMT0068B3<br>MGG_00138.6 T-DNA 500 bp	TF041	Reduced	Normal	>50%	No App.	-
ATMT0349D2<br>T-DNA 1369 bp MGG_02755.6	TF031	Reduced	no record	>50%	>50%	Normal
ATMT0052B2<br>MGG_07218.6 T-DNA 810 bp	TF233	Normal	Abnormal	<50%	<50%	No disease
ATMT0591D1<br>T-DNA MGG_09263.6	TF260	Normal	Abnormal	>50%	<50%	Reduced
ATMT0034B1<br>T-DNA 1283 bp MGG_06507.6	TF150	Normal	Abnormal	>50%	>50%	No disease

Figure S5. T-DNA insertion sites in eight mutants defective in conidiation-specific TF genes and resulting phenotypes. Phenotypes of these mutants are derived from *Magnaporthe oryzae* T-DNA insertion mutant library (http://atmt.snu.ac.kr).
