# Supplementary material for: Global Expression Profiling of Transcription Factor Genes Provides New Insights into Pathogenicity and Stress Responses in the Rice Blast Fungus
Source: PLoS Pathog. 2013 Jun 6;9(6):e1003350. doi: 10.1371/journal.ppat.1003350 (PMC3675110; doi:10.1371/journal.ppat.1003350)
Supplement: Figure S6 — Conidiation at four different time points. (A) Pictures of fungal culture on cellulose nitrate membrane filter laid with on V8-Juice agar medium from 0 h to 24 h. (B) Numbers of conidia (per plate) at the indicated time. (C) Diagram illustrating conidiation process based on microscopic observation. Three genes involved at different stages of conidition, which are based on a study by Liu et al. (2010), are noted. (D) Expression patterns of these three genes during conidiation and/or in conidia in wild-type KJ201 are shown. (PDF) [file ppat.1003350.s006.pdf]

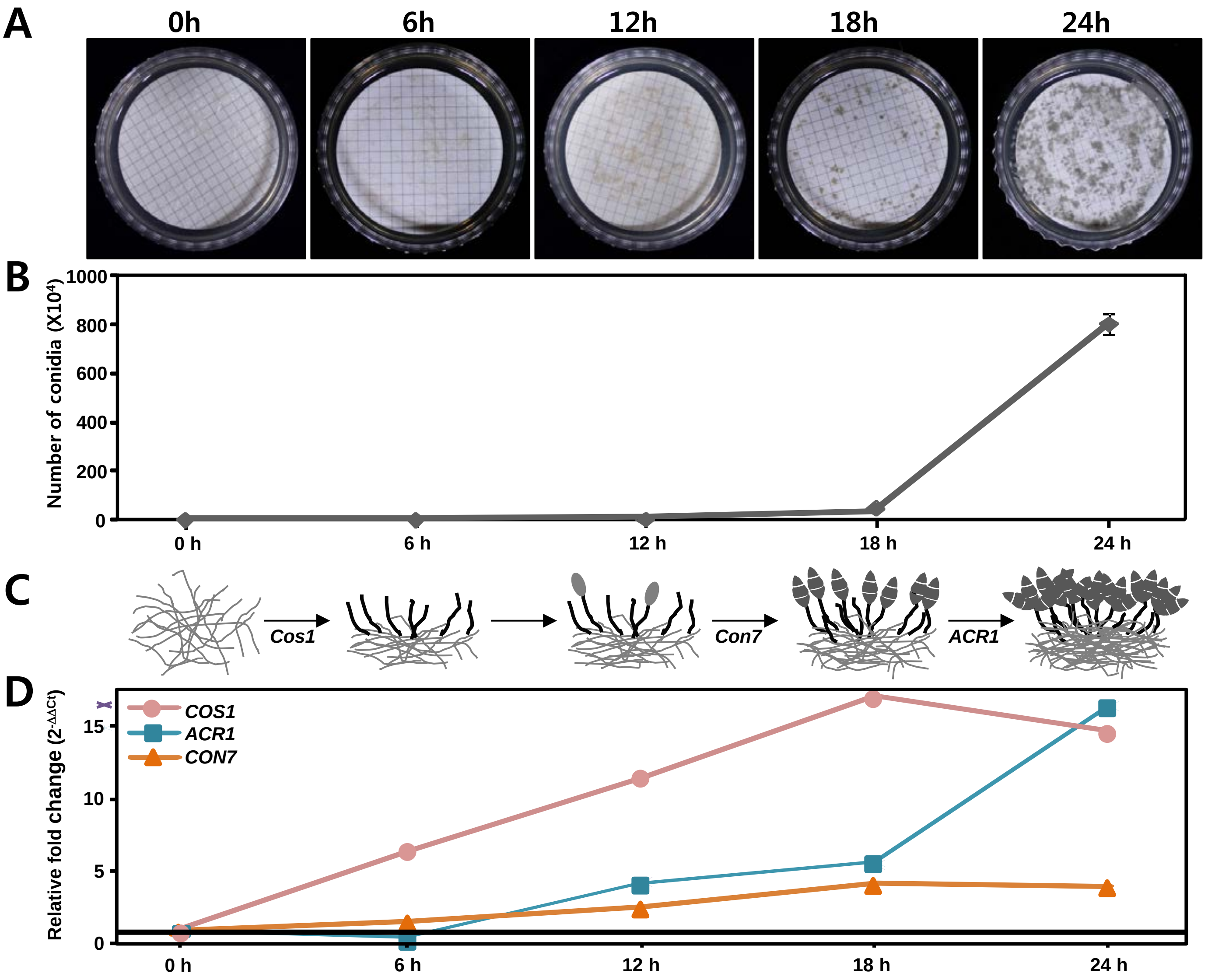

Figure S6. Conidiation at four different time points. (A) Pictures of fungal culture on cellulose nitrate membrane filter laid with on V8-Juice agar medium from 0h to 24h. (B) Numbers of conidia (per plate) at the indicated time. (C) Diagram illustrating conidiation process based on microscopic observation. Three genes involved at different stages of conidition, which are based on a study by Liu *et al*. (2010), are noted. (D) Expression patterns of these three genes during conidiation and/or in conidia in wild-type KJ201 are shown.

Reference

Liu W, Xie S, Zhao X, Chen X, Zheng W, et al. (2010) A homeobox gene is essential for conidiogenesis of the rice blast fungus *Magnaporthe oryzae*. Mol Plant Microbe Interact 23: 366-375.
