# Supplementary material for: Global Expression Profiling of Transcription Factor Genes Provides New Insights into Pathogenicity and Stress Responses in the Rice Blast Fungus
Source: PLoS Pathog. 2013 Jun 6;9(6):e1003350. doi: 10.1371/journal.ppat.1003350 (PMC3675110; doi:10.1371/journal.ppat.1003350)
Supplement: Table S1 — Transcription factors encoded by 23 fungal and Oomycete genomes. (PDF) [file ppat.1003350.s007.pdf]

Table S1. Transcription factors encoded by 23 fungal and Oomycete genomes

**Species name**	**Phylum/ Subphylum**	**No. of ORFs**	**No. of TF families**	**No. of TFs**	**Ratio**	**Ref.***
Magnaporthe oryzae	Pezizomycotina	11,069	44	495	4.49%	(1)
Fusarium graminearum	Pezizomycotina	13,339	44	659	4.94%	(2)
Fusarium oxysporum	Pezizomycotina	17,735	43	795	4.48%	NA
Neurospora crassa	Pezizomycotina	9,842	44	420	4.27%	(3)
Podospora anserina	Pezizomycotina	10,596	41	477	4.50%	(4)
Stagonospora nodorum	Pezizomycotina	15,893	39	442	2.77%	(5)
Uncinocarpus reesii	Pezizomycotina	7,798	44	321	4.12%	
Aspergillus fumigatus	Pezizomycotina	9,887	44	562	5.68%	(6)
Aspergillus nidulans	Pezizomycotina	10,568	44	608	5.75%	(7)
Yarrowia lipolytica	Saccharomycotina	6,524	42	289	4.43%	(8)
Saccharomyces cerevisiae	Saccharomycotina	6,700	36	295	4.41%	(9)
Candida glabrata	Saccharomycotina	5,165	38	262	5.07%	(8)
Debaryomyces hansenii	Saccharomycotina	6,354	42	324	5.10%	(8)
Candida albicans	Saccharomycotina	6,185	39	317	5.13%	(10, 11)
Schizosaccharomyces pombe	Taphrinomycotina	5,058	38	222	4.39%	(12)
Cryptococcus neoformans	Basidiomycota	6,475	34	307	4.74%	(13)
Phanerochaete chrysosporium	Basidiomycota	10,048	37	387	3.85%	(14)
Laccaria bicolor	Basidiomycota	20,614	39	494	2.40%	(15)
Ustilago maydis	Basidiomycota	6,689	40	331	4.95%	(16)
Rhizopus oryzae	Mucoromycotina	17,482	41	1,110	6.35%	(17)
Encephalitozoon cuniculi	Microsporodia	1,996	22	108	5.41%	(18)
Phytophthora sojae	Oomycetes	19,027	42	919	4.83%	(19)
Phytophthora ramorum	Oomycetes	15,743	40	641	4.07%	(19)
**Total**		240,787		10,785	4.46%	-

*** References**

1. Dean RA, Talbot NJ, Ebbole DJ, Farman ML, Mitchell TK, Orbach MJ, Thon M, Kulkarni R, Xu JR, Pan H, *et al.* (2005) *Nature* **434,** 980-986.
2. Cuomo CA, Guldener U, Xu JR, Trail F, Turgeon BG, Di Pietro A, Walton JD, Ma LJ, Baker SE, Rep M, *et al.* (2007) *Science* **317,** 1400-1402.
3. Galagan JE, Calvo SE, Borkovich KA, Selker EU, Read ND, Jaffe D, FitzHugh W, Ma LJ, Smirnov S, Purcell S, *et al.* (2003) *Nature* **422,** 859-868.
4. Espagne E, Lespinet O, Malagnac F, Da Silva C, Jaillon O, Porcel BM, Couloux A, Aury JM, Segurens B, Poulain J, *et al.* (2008) *Genome Biol* **9,** R77.
5. Hane JK, Lowe RG, Solomon PS, Tan KC, Schoch CL, Spatafora JW, Crous PW, Kodira C, Birren BW, Galagan JE, *et al.* (2007) *Plant Cell* **19,** 3347-3368.
6. Nierman WC, Pain A, Anderson MJ, Wortman JR, Kim HS, Arroyo J, Berriman M, Abe K, Archer DB, Bermejo C, *et al.* (2005) *Nature* **438,** 1151-1156.
7. Galagan JE, Calvo SE, Cuomo C, Ma LJ, Wortman JR, Batzoglou S, Lee SI, Basturkmen M, Spevak CC, Clutterbuck J, *et al.* (2005) *Nature* **438,** 1105-1115.
8. Dujon B, Sherman D, Fischer G, Durrens P, Casaregola S, Lafontaine I, De Montigny J, Marck C, Neuveglise C, Talla E, *et al.* (2004) *Nature* **430,** 35-44.
9. Goffeau A, Barrell BG, Bussey H, Davis RW, Dujon B, Feldmann H, Galibert F, Hoheisel JD, Jacq C,

Johnston M, *et al.* (1996) *Science* **274,** 546, 563-547.
10. Jones T, Federspiel NA, Chibana H, Dungan J, Kalman S, Magee BB, Newport G, Thorstenson YR, Agabian N, Magee PT, *et al.* (2004) *Proc Natl Acad Sci USA* **101,** 7329-7334.
11. van het Hoog M, Rast TJ, Martchenko M, Grindle S, Dignard D, Hogues H, Cuomo C, Berriman M, Scherer S, Magee BB, *et al.* (2007) *Genome Biol* **8,** R52.
12. Wood V, Gwilliam R, Rajandream MA, Lyne M, Lyne R, Stewart A, Sgouros J, Peat N, Hayles J, Baker S, *et al.* (2002) *Nature* **415,** 871-880.
13. Loftus BJ, Fung E, Roncaglia P, Rowley D, Amedeo P, Bruno D, Vamathevan J, Miranda M, Anderson IJ, Fraser JA, *et al.* (2005) *Science* **307,** 1321-1324.
14. Martinez D, Larrondo LF, Putnam N, Gelpke MD, Huang K, Chapman J, Helfenbein KG, Ramaiya P, Detter JC, Larimer F, *et al.* (2004) *Nat Biotechnol* **22,** 695-700.
15. Martin F, Aerts A, Ahren D, Brun A, Danchin EG, Duchaussoy F, Gibon J, Kohler A, Lindquist E, Pereda V, *et al.* (2008) *Nature* **452,** 88-92.
16. Kamper J, Kahmann R, Bolker M, Ma LJ, Brefort T, Saville BJ, Banuett F, Kronstad JW, Gold SE, Muller O, *et al.* (2006) *Nature* **444,** 97-101.
17. Ma LJ, Ibrahim AS, Skory C, Grabherr MG, Burger G, Butler M, Elias M, Idnurm A, Lang BF, Sone T, *et al.* (2009) *PLoS Genet* **5,** e1000549.
18. Katinka MD, Duprat S, Cornillot E, Metenier G, Thomarat F, Prensier G, Barbe V, Peyretaillade E, Brottier P, Wincker P, *et al.* (2001) *Nature* **414,** 450-453.
19. Tyler BM, Tripathy S, Zhang X, Dehal P, Jiang RH, Aerts A, Arredondo FD, Baxter L, Bensasson D, Beynon JL, *et al.* (2006) *Science* **313,** 1261-1266.
