# Supplementary material for: Global Expression Profiling of Transcription Factor Genes Provides New Insights into Pathogenicity and Stress Responses in the Rice Blast Fungus
Source: PLoS Pathog. 2013 Jun 6;9(6):e1003350. doi: 10.1371/journal.ppat.1003350 (PMC3675110; doi:10.1371/journal.ppat.1003350)
Supplement: Table S2 — Distribution of putative homologues of 26 Magnaporthe oryzae -specific transcription factor genes analyzed using Blast Matrix and InParanoid algorism. (PDF) [file ppat.1003350.s008.pdf]

Table S2. Distribution of putative homologues of 26 *Magnaporthe oryzae* -specific transcription factor genes analyzed using Blast Matrix and InParanoid algorism

TF families	Protein Name																																		No orthologe found by InParanoid
	Kingdom	Chromista	Fungi																											Metazoa			Viridiolantae		
	Phylum	Oomycota	Ascomycota																Basidiomycota						Blastocladiomycota	Chytridiomycota	Microsporidia	Zygomycota		Arthropoda	Chordata	Nematoda	Streptoph		
	Class	Oomycetes	Saccharomycetes		Schizosaccharomycetes	Eurotiomycetes		Eurotiomycetes		Leotiomycetes	Sordariomycetes								Aearicomycetes			Heterobasidiomycetes	Not defined	Ustilagomycetes	Blastocladiomycetes	Not defined				Insecta	Mammalia	Chromadorea	Liliopsida	Eurosids II	
	Species	Pi	Cas	Sc	Sp	Mgr	Sn	Af1	An	Bc	Fg	Fol	Fs	Mo	Nc	Pa	Tr	Vd	Ds	Ha	Pc	Cn	Pg	Um5	Am	BdJ	Ecu	Pbl	Ro	Dm	Hs	Ce	Osj	Ath	
Zn2Cys6	MGG_00021.6	-	-	-	-	-	-	-	-	-	-	-	-	±	-	-	-	-	-	-	-	-	-	-	-	-	-	-	-	-	-	-	-	-	MGG_00021.6
	MGG_02600.6	-	±	±	±	±	±	±	±	±	±	±	±	±	±	±	±	±	±	±	±	±	-	±	-	±	-	±	±	-	-	-	-	-	MGG_02600.6
	MGG_04326.6	-	-	-	-	±	±	±	±	±	±	±	±	±	-	±	±	±	-	±	-	±	-	±	-	-	-	-	-	-	-	-	-	-	MGG_04326.6
	MGG_04360.6	-	-	-	±	-	±	±	-	±	±	±	±	±	±	-	±	±	-	-	-	-	-	-	±	-	-	-	±	-	-	-	-	-	MGG_04360.6
	MGG_05845.6	-	-	-	-	-	-	-	-	-	-	-	±	±	-	-	-	±	-	-	-	-	-	-	-	-	-	-	-	-	-	-	-	-	MGG_05845.6
MHG	MGG_02978.6	-	±	±	±	±	±	±	±	±	±	±	±		±	±	±	±	±	±	±	±	±	±	±	±	-	±	±	±	±	±	-	±	MGG_02978.6
	MGG_02979.6	-	±	±	±	-	-	±	-	±	±	±	±		±	±	±	±	±	±	±	±	±	±	±	±	-	±	±	±	±	-	-	-	MGG_02979.6
	MGG_04570.6	±	-	-	-	±	-	±	±	±	±	±	±		±	±	±	±	±	±	±	±	±	±	±	±	-	-	±	±	±	±	±	±	MGG_04570.6
	MGG_05369.6	±	-	-	-	±	-	±	-	±	±	±	±		±	±	-	±	-	±	±	±	±	±	-	±	-	±	-	-	±	±	-	±	MGG_05369.6
	MGG_07057.6	-	-	±	-	-	±	±	±	±	±	±	±		±	±	±	±	±	±	±	±	±	±	±	-	-	-	±	±	±	±	±	±	MGG_07057.6
	MGG_07674.6	±	±	±	±	±	±	±	±	±	±	±	±		±	±	±	±	±	±	±	±	±	±	±	±	±	±	±	±	±	±	-	±	MGG_07674.6
	MGG_09739.6	±	-	-	±	±	±	±	±	-	±	±	±		±	±	±	±	±	±	±	±	±	±	±	-	-	±	±	±	±	±	±	±	MGG_09739.6
	MGG_13742.6	±	±	±	±	±	±	±	±	±	±	±	±		±	±	±	±	-	±	±	±	±	±	±	±	-	±	±	±	±	±	-	±	MGG_13742.6
	MGG_15309.6	-	-	-	±	±	±	±	±	-	-	±	±		±	±	±	±	±	±	±	±	±	-	±	-	-	±	-	-	±	-	±	±	MGG_15309.6
C2H2	MGG_14358.6	-	-	-	-	-	-	-	±	±	±	±	-		±	-	±	±	-	-	-	±	-	-	-	-	-	-	-	-	-	±	-	-	MGG_14358.6
Homeodomain-like	MGG_09628.6	-	-	-	-	-	-	-	-	-	-	-	-		-	-	-	-	-	-	-	-	-	-	-	-	-	-	-	-	-	-	-	-	MGG_09628.6
	MGG_14987.6	-	-	-	-	-	-	-	-	-	-	±	±		-	-	-	-	-	-	-	-	-	-	-	-	-	-	-	-	-	-	-	-	MGG_14987.6
CCHC	MGG_01763.6	-	-	-	±	-	±	-	-	±	-	-	-		±	-	-	±	-	-	-	-	-	-	±	±	-	±	-	-	-	-	-	±	MGG_01763.6
	MGG_08239.6	±	±	±	±	±	±	-	±	±	±	±	±		±	±	±	±	±	±	±	±	±	±	±	±	-	±	±	±	±	±	±	±	MGG_08239.6
	MGG_15341.6	-	-	-	-	-	-	-	-	-	-	-	-		-	-	-	-	-	-	-	-	-	-	-	-	-	-	-	-	-	-	-	-	MGG_15341.6
Winged helix repressor	MGG_15412.6	-	-	-	-	-	-	-	-	-	-	-	-		-	-	-	-	-	-	-	-	-	-	-	-	-	-	-	-	-	-	-	-	MGG_15412.6
	MGG_03277.6	-	-	-	-	-	-	-	-	-	-	-	-		±	±	-	-	-	-	-	-	-	-	-	-	-	-	-	-	-	-	-	-	MGG_03277.6
GATA	MGG_07423.6	-	-	-	-	-	-	-	-	-	-	-	-		-	-	±	-	-	-	-	-	-	-	-	-	-	-	-	-	-	-	-	-	MGG_07423.6
	MGG_10970.6	-	±	±	-	±	±	±	±	±	±	±	±		±	±	±	±	±	±	±	±	±	±	±	±	±	±	±	-	-	-	±	±	MGG_10970.6
GRF-type	MGG_08489.6	-	-	-	-	±	-	-	-	±	±	±	±		±	±	±	±	-	±	±	-	-	±	±	-	-	-	-	-	-	-	-	-	MGG_08489.6
mbda repressor-l	MGG_11615.6	±	±	±	±	±	±	±	±	±	±	±	±		±	±	±	±	±	±	±	±	±	±	±	±	-	±	±	±	±	±	±	±	MGG_11615.6

: 1e-100 ; ± : 1e-50 ; ± : 1e-30 ; ± : 1e-10 ; - : None

*Abbreviation for fungal species and other organisms: Pi: *Phytophthora infestans* ; Cas: *Candida albicans* SC5314; Sc: *Saccharomyces cerevisiae* S288C; Sp: *Schizosaccharomyces pombe* ; Mgr: *Mycosphaerella graminicola* ; Sn: *Stagonospora nodorum* ; Af: *Aspergillus fumigatus* Af293; An: *Aspergillus nidulans* 4; Bc: *Botrytis cinerea* ; Fg: *Fusarium graminearum* ; Fol: *Fusarium oxysporum* ; Fs: *Nectria haematococca* (*F. solani*) ; Mo: *Magnaporthe oryzae* 70-15; Nc: *Neurospora crassa* ; Pa: *Podospora anserina* ; Tr: *Trichoderma reesei* ; Vd: *Verticillium dahliae* VdLs.17; Ds: *Dichomitus squalens* ; Ha: *Heterobasidion annosum* ; Pc: *Phanerochaete chrysosporium* ; Cn: *Cryptococcus neoformans* variety *gatti* (serotype A) ; Pg: *Puccinia graminis* ; Um: *Ustilago maydis* 521; Am: *Allomyces macrogynus* ; Bdj: *Batrachochytrium dendrobatidis* JAM81; Ec: *Encephalitozoon cuniculi* ; Pbl: *Phycomyces blakesleeanus* ; Ro: *Rhizopus oryzae* ; Dm: *Dorosophila melanogaster* ; Hs: *Homo sapiens* ; Ce: *Caenorhabditis elegans* ; Osj: *Oryza sativa* japonica; Ath: *Arabidopsis thaliana* .
