# Supplementary material for: Global Expression Profiling of Transcription Factor Genes Provides New Insights into Pathogenicity and Stress Responses in the Rice Blast Fungus
Source: PLoS Pathog. 2013 Jun 6;9(6):e1003350. doi: 10.1371/journal.ppat.1003350 (PMC3675110; doi:10.1371/journal.ppat.1003350)
Supplement: Table S3 — Primers used to amplify potential reference genes needed for gene expression analyses. (PDF) [file ppat.1003350.s009.pdf]

Table S3. Primers used to amplify potential reference genes needed for gene expression analyses

Gene symbol*	Gene locus version 6	Forward primer (5'-3')	Reverse primer (5'-3')	Amplicon (bp)
β-tubulin	MGG_00604.6	ACAACTTCGTCTTCGGTCAG	GTGATCTGGAAACCCTGGAG	203 bp
α-tubulin	MGG_06650.6	GCTGTCTGCGTTCTCTCC	TACTCCGCCTCAATACCCT	230 bp
EF1β	MGG_04436.6	AAGCCTTGGGATGATGAGAC	AGCGACATCGGTGGACTGG	204 bp
UEP1	MGG_06044.6	CTCTGGCCAGCAAGTTCAAC	CGCCAAAGACCGTGATG	167 bp
Actin2	MGG_03982.6	CTATCAACCCCAAGTCCAAC	CAAGGAGAAACCCTCGTAAA	188 bp
GAPDH	MGG_01084.6	TATCAACGGTTTCGGTCGTA	AACTTGACCTTCTTGCCGTT	207 bp
CYP1	MGG_10447.6	GCCTAACGTTTTCTTCGACATTTC	GTTCTCGTCGGCAAACTTCTC	253 bp

**EF1β*: elongation factor1-*β*; *UEP1*: ubiquitin extension protein gene; *GAPDH*: glyceraldehydes-3-phosphate dehydrogenase; *CYP1*: cyclophilin.
