# Supplementary material for: Global Expression Profiling of Transcription Factor Genes Provides New Insights into Pathogenicity and Stress Responses in the Rice Blast Fungus
Source: PLoS Pathog. 2013 Jun 6;9(6):e1003350. doi: 10.1371/journal.ppat.1003350 (PMC3675110; doi:10.1371/journal.ppat.1003350)
Supplement: Table S4 — Conditions used to extract RNA samples for gene expression analyses. (PDF) [file ppat.1003350.s010.pdf]

Table S4. Conditions used to extract RNA samples for gene expression analyses

**Categories**	**Condition**	**Conc.**	**Treated time**	**Basal media for treatment ***	**Abbreviation**
Cell development	Conidia	-			Con
	Conidial gemlings	-			Ger
	Appressoria	-			App
	78 hpi compatible rice †	-	78 hpi		78hpi
	150 hpi compatible rice	-	150 hpi		150hpi
Oxidative stress	H_2O_2	1mM	4 hr	CM	Px1
	H_2O_2	5mM	4 hr	CM	Px5
	Methyl viologen	10mM	4 hr	CM	MV
Nutrient utilization and uptake	Fully fed	-	4 hr	CM	CM
	Thiamine	100mM	4 hr	CM	Thi
	Minimal medium	-	4 hr	MM	MM
	C-starvation	-	4 hr	CM	-C
	N-starvation	-	4 hr	CM	-N
	Uric acid	5mM	4 hr	MM	UA
	KH_2PO_4	7.35mM	4 hr	MM	K7.35
	KH_2PO_4	0.05mM	4 hr	MM	K0.05
Ambient pH	pH 4	-	4 hr	MM	pH4
	pH 8	-	4 hr	MM	pH8
Ionic stresses	LiCl	0.1M	4 hr	MM	Li
	NaCl	1M	4 hr	MM	Na
	$FeSO_4$	1mM	4 hr	MM	Fe1
	$FeSO_4$	10mM	4 hr	MM	Fe10
	$MnCl_2$	10mM	4 hr	CM	Mn
	$CuSO_4$	4mM	4 hr	CM	Cu
DNA repair	UV irradiation	-	40 sec.	CM	UVS
	UV irradiation	-	4 min.	CM	UVL
Phenolic compound	*p*-coumaric acid	150ppm	4 hr	CM	PC
Phenolic compound	Acetosyringone	20mM	4 hr	CM	AS
Catalase inhibitor	3-Amino-1,2,4-triazole	50mM	4 hr	CM	3AT
Cell wall stress	Congo Red	100ppm	4 hr	CM	CR
Microtubule binding	Benomyl	20ppm	4 hr	CM	Beno
Heat shock stress	Heat Shock 42 °C	-	45 min	CM	Heat

* Analysis of relative expression was calculated by expression under complete and minimal media used as basal medium.
† cv. Nakdongbyeo has not contained any known resistant gene toward the rice blast fungus.
