# Supplementary material for: Global Expression Profiling of Transcription Factor Genes Provides New Insights into Pathogenicity and Stress Responses in the Rice Blast Fungus
Source: PLoS Pathog. 2013 Jun 6;9(6):e1003350. doi: 10.1371/journal.ppat.1003350 (PMC3675110; doi:10.1371/journal.ppat.1003350)
Supplement: Table S5 — List of TF genes up-regulated under developmental conditions. (PDF) [file ppat.1003350.s011.pdf]

Table S5. List of TFs up-regulated under developmental conditions

	TF Family	Locus	TF name	Previously studied TFs	Homologues gene (Putative function)
Conidiation	APSES	MGG_08463.6	TF010	*MoAPS2*	*Swi6* (regulation of cell cycle progression from G1 to S phase) in *S. cerevisiae*
	APSES	MGG_09869.6	TF011	*MoAPS1*	-
	bHLH	MGG_01321.6	TF003		-
	bHLH	MGG_10837.6	TF008		white collar-2 in *N. crassa*; sexual development transcription factor in *A. nidulans*
	Forkhead	MGG_01853.5	FOK1		A minor role in the expression of G2/M phase genes in *S. cerevisiae*
	Forkhead	MGG_06422.5	FOK3		Chromatin modification-related protein vid-21 in *P. ancerina*
	Homeobox	MGG_04853.6	HOX1	*MoHOX1*	tRNA isopentenyltransferase in *A. Nidulans*
	Homeobox	MGG_00184.6	HOX2	*MoHOX2*	-
	Homeobox	MGG_01730.6	HOX3	*MoHOX3*	*PTR2* (transcriptional repressor) in *S. cerevisiae*
	Homeobox	MGG_06285.6	HOX4	*MoHOX4*	-
	Homeobox	MGG_11712.6	HOX6	*MoHOX6*	-
	Homeobox	MGG_12958.6	HOX8	*MoHOX8*	TF that activated by a MAP kinase signaling cascade in *S. cerevisiae*
	Myb	MGG_00138.6	TF041		-
	Myb	MGG_01012.6	TF042		Chromatin remodelling complex ATPase chain ISW1 in *N. crassa*
	Myb	MGG_01426.6	TF044		nitrate nonutilizer-4 in *N. crassa*; nitrogen assimilation TF *nirA* in *A. nidulans*.
	Myb	MGG_02746.6	TF046		nitrate nonutilizer-2 in *N. crassa*; regulatory protein areA in *A. nidulans.*
	Myb	MGG_05099.6	TF048		Transcription coactivator in *S. cerevisiae*
	Myb	MGG_05240.6	TF049		Regulation of basal and induced expression in *S. cerevisiae*
	Myb	MGG_05748.6	TF050		-
	Myb	MGG_06434.6	TF053		*TamA* in *N. crassa*
	GATA	MGG_02755.6	TF031		Transcriptional activator for nitrogen catabolite repression in *S. cerevisiae*
	GATA	MGG_07319.6	TF035		*Gln3p* and *Dal80p*; expression repressed by leucine in *S. cerevisiae*
	C2H2	MGG_00139.6	TF058		E3 ubiquitin-protein ligase *BRE1*
	C2H2	MGG_01171.6	TF059		-
	C2H2	MGG_09837.6	TF068		*Swi6* in *N. crassa*, *A. nidulans*, *P. anserina*, and *F. graminearum*
	C2H2	MGG_00702.6	TF070		-
	C2H2	MGG_02447.6	TF073		-
	C2H2	MGG_08340.6	TF085		-
	C2H2	MGG_00373.6	TF094		-
	C2H2	MGG_02474.6	TF116		Activates expression of early G1-specific genes in *S. cerevisiae*
	C2H2	MGG_02505.6	TF117		-
	C2H2	MGG_11252.6	TF133		-
	C2H2	MGG_02845.6	TF134		*xlnR* (transcriptional activator) in *N. crassa* and *P. anserina*
	C2H2	MGG_06507.6	TF150		Induction of *CLN3* transcription in response to glucose in *S. cerevisiae*
	C2H2	MGG_08114.6	TF155		-
	Zn2Cys6	MGG_00672.6	TF165	*MoLEU3*	-
	Zn2Cys6	MGG_00934.6	TF166		Glucose-responsiveTF in *S. cerevisiae*
	Zn2Cys6	MGG_01518.6	TF169	*MoNIT4*	Arsenite resistance protein *Ars2* in *N. crassa*
	Zn2Cys6	MGG_01777.6	TF173		Lactose regulatory protein *LAC9* in *P. ancerina*
	Zn2Cys6	MGG_01833.6	TF175		Siderophore regulation in *N. crassa*; *SREA* in *A. nidulans*.
	Zn2Cys6	MGG_02377.6	TF180		-
	Zn2Cys6	MGG_03711.6	TF192		Transcriptional activator for derepression of a variety of genes in *S. cerevisiae*
	Zn2Cys6	MGG_04951.6	TF206		-
	Zn2Cys6	MGG_06243.6	TF220		-
	Zn2Cys6	MGG_06279.6	TF221		-
	Zn2Cys6	MGG_07063.6	TF230		-
	Zn2Cys6	MGG_07131.6	TF231		Regulation of lysine biosynthesis pathway in *S. cerevisiae*; *RfeC* in *N. crassa*.
	Zn2Cys6	MGG_07218.6	TF233		Activation of the *GAL* genes in response to galactose in *S. cerevisiae*
	Zn2Cys6	MGG_07681.6	TF241		-
	Zn2Cys6	MGG_08094.6	TF246		*SWR1*-complex protein 4 in *N. crassa.*
	Zn2Cys6	MGG_09263.6	TF260		-
	Zn2Cys6	MGG_09312.6	TF263		Activation of the *GAL* genes in response to galactose in *S. cerevisiae*
	Zn2Cys6	MGG_12037.6	TF264		-
	Zn2Cys6	MGG_09825.6	TF268		-
	Zn2Cys6	MGG_09829.6	TF269		Vacuolar segregation protein pep7 in *N. crassa*.
	Zn2Cys6	MGG_09950.6	TF271		pH-response transcription factor *pacC/RIM101* in *N. crassa*.
	Zn2Cys6	MGG_10528.6	TF276		-
Germination	bHLH	MGG_00595.6	TF001		-
	bHLH	MGG_10575.6	TF007		-
	C2H2	MGG_01127.6	TF142		Stimulates expression of proteasome genes in *S. cerevisiae*

	Myb	MGG_06120.6	TF052		UBX domain-containing protein in *N. crassa*
	Myb	MGG_08137.6	TF056		-
Appressroium formation	C2H2	MGG_09035.6	TF086		-
	C2H2	MGG_05518.6	TF078		-
	Zn2Cys6	MGG_01946.6	TF178		-
	Zn2Cys6	MGG_15023.6	TF184		-
	Zn2Cys6	MGG_15346.6	TF253		-
	Zn2Cys6	MGG_06954.6	TF229		Sterol regulatory element binding protein in *S. cerevisiae*
	Zn2Cys6	MGG_15085.6	TF234		Transcriptional regulator involved in the stress response in *S. cerevisiae*
	Zn2Cys6	MGG_09276.6	TF262		-
All the conditions	bHLH	MGG_05709.6	TF006		-
	C2H2	MGG_00076.6	TF069		*Sip-5* in *N. crassa*
	C2H2	MGG_03581.6	TF118		-
	C2H2	MGG_02775.6	TF100		Transcriptional regulation of the methionine biosynthetic genes in *S. cerevisiae*
	C2H2	MGG_04699.6	TF101		*kaleidoscope-1* in *N. crassa* .
	C2H2	MGG_10280.6	TF090		-
	C2H2	MGG_07011.6	TF104		-
	C2H2	MGG_08015.6	TF106		-
	C2H2	MGG_09701.6	TF131		Melanization defective-1 in *N. crassa*
	C2H2	MGG_05714.6	TF154		Activates expression of early G1-specific genes in *S. cerevisiae*
	Homeobox	MGG_12865.6	HOX7	*MoHox7*	protoperithecium-1 in *N. crassa* ; *SteA* in *A. nidulans*
	Myb	MGG_06898.6	TF054		*APF1* in *N. crassa* ; antigenic determinant of *recA* protein in *A. nidulans*
	Zn2Cys6	MGG_01285.6	TF167		-
	Zn2Cys6	MGG_05343.6	TF210		-
	Zn2Cys6	MGG_00320.6	TF161		Acriflavine sensitivity control protein *acr-2*
	Zn2Cys6	MGG_01624.6	TF170		-
	Zn2Cys6	MGG_14900.6	TF199		Telomere uncapping and elongation as component in *S. cerevisiae*
	Zn2Cys6	MGG_05939.6	TF218		-
	Zn2Cys6	MGG_10197.6	TF272		-
	Zn2Cys6	MGG_10422.6	TF275		-
	Zn2Cys6	MGG_00021.6	TF158		-
	Zn2Cys6	MGG_00049.6	TF159		-
	Zn2Cys6	MGG_02880.6	TF186		-
	Zn2Cys6	MGG_06492.6	TF226		Positive regulator in multiple nitrogen degradation pathways in *S. cerevisiae*
	Zn2Cys6	MGG_06832.6	TF228		-
	Zn2Cys6	MGG_07845.6	TF244		*RING-6* in *N. crassa* and *F. graminearum*
	Zn2Cys6	MGG_08199.6	TF249		-
	Zn2Cys6	MGG_08314.6	TF250		Unknown function that may interact with ribosomes in *S. cerevisiae*
	Zn2Cys6	MGG_08753.6	TF255		-
	Zn2Cys6	MGG_08917.6	TF257		Transcriptional activator of proline utilization genes in *S. cerevisiae*
	Zn2Cys6	MGG_10802.6	TF278		Positively regulates transcription in *S. cerevisiae*

: *M. oryzae* specific TFs

: Reduction of conidia production by T-DNA insertion mutants

: Production of abnormal conidia by T-DNA insertion mutants

: No conidia production
