# Supplementary material for: Global Expression Profiling of Transcription Factor Genes Provides New Insights into Pathogenicity and Stress Responses in the Rice Blast Fungus
Source: PLoS Pathog. 2013 Jun 6;9(6):e1003350. doi: 10.1371/journal.ppat.1003350 (PMC3675110; doi:10.1371/journal.ppat.1003350)
Supplement: Table S6 — Relative abundance of transcripts from the 57 conidiation-specific TF genes during conidiation and/or in conidia. (PDF) [file ppat.1003350.s012.pdf]

Table S6. Relative abundance of transcripts from the 57 conidiation-specific TF genes during conidiation and /or in conidia

Family	Locus	No. TF	Fold change				
			0h	6h	12h	18h	24h
GATA	MGG_07319.6	TF035	1	4.0	10.3	10.7	8.5
C2H2	MGG_00139.6	TF058	1	2.4	3.3	4.0	3.1
C2H2	MGG_02447.6	TF073	1	2.9	3.4	4.3	3.3
Zn2Cys6	MGG_07681.6	TF241	1	2.8	3.4	3.5	3.6
Zn2Cys6	MGG_09263.6	TF260	1	2.6	4.7	4.0	3.4
Zn2Cys6	MGG_01833.6	TF175	1	2.2	3.5	2.8	4.3
Zn2Cys6	MGG_06243.6	TF220	1	2.3	3.8	3.6	4.8
Homeobox	MGG_01730.6	HOX3	1	7.0	3.4	2.7	1.9
C2H2	MGG_02505.6	TF117	1	3.2	2.9	3.2	1.9
Zn2Cys6	MGG_06279.6	TF221	1	3.3	3.3	6.5	1.6
Homeobox	MGG_11712.6	HOX6	1	1.9	2.9	2.9	2.3
Myb	MGG_00138.6	TF041	1	1.9	2.6	2.6	2.8
Myb	MGG_05099.6	TF048	1	1.5	3.0	3.1	3.4
Myb	MGG_06434.6	TF053	1	1.8	3.2	3.3	3.0
C2H2	MGG_01171.6	TF059	1	1.5	2.3	2.4	2.3
C2H2	MGG_09837.6	TF068	1	1.4	2.2	2.5	2.1
C2H2	MGG_00373.6	TF094	1	1.5	2.7	2.6	2.1
C2H2	MGG_08114.6	TF155	1	1.4	2.0	2.0	2.1
Zn2Cys6	MGG_01777.6	TF173	1	1.9	3.1	3.0	2.7
Zn2Cys6	MGG_02377.6	TF180	1	1.3	2.2	3.7	2.6
Zn2Cys6	MGG_04951.6	TF206	1	1.8	3.2	3.4	2.8
Zn2Cys6	MGG_09829.6	TF269	1	1.1	5.8	6.1	14.4
Zn2Cys6	MGG_09950.6	TF271	1	0.9	2.5	2.0	2.3
Zn2Cys6	MGG_10528.6	TF276	1	1.5	2.5	2.5	2.5
Homeobox	MGG_04853.6	HOX1	1	1.7	2.3	3.0	1.6
Homeobox	MGG_00184.6	HOX2	1	1.4	2.2	2.3	1.6
Homeobox	MGG_06285.6	HOX4	1	1.4	2.1	2.2	1.5
APSES	MGG_08463.6	TF010	1	1.2	2.4	3.0	1.1
APSES	MGG_09869.6	TF011	1	1.7	2.0	2.7	1.1
Myb	MGG_01012.6	TF042	1	1.7	2.3	3.2	1.8
Myb	MGG_05748.6	TF050	1	1.2	2.4	2.4	1.8
C2H2	MGG_08340.6	TF085	1	1.6	2.0	2.0	1.7
C2H2	MGG_02845.6	TF134	1	1.0	2.0	2.0	1.8
bHLH	MGG_01321.6	TF003	1	1.2	1.9	2.2	2.4
Zn2Cys6	MGG_00672.6	TF165	1	2.0	1.3	1.6	1.2
Zn2Cys6	MGG_07218.6	TF233	1	1.0	2.0	1.6	1.3
Zn2Cys6	MGG_08094.6	TF246	1	1.5	2.2	2.1	1.8
Zn2Cys6	MGG_09312.6	TF263	1	1.2	2.1	2.2	1.8
Forkhead	MGG_01853.5	FOK1	1	1.0	1.5	2.0	1.4
Homeobox	MGG_12958.6	HOX8	1	1.3	1.7	2.2	1.0
bHLH	MGG_10837.6	TF008	1	1.0	1.9	2.0	1.7
Myb	MGG_01426.6	TF044	1	1.4	1.9	2.2	1.4
Myb	MGG_05240.6	TF049	1	0.9	1.8	3.2	1.9
C2H2	MGG_06507.6	TF150	1	0.6	1.3	2.0	0.7
Zn2Cys6	MGG_00934.6	TF166	1	1.8	1.6	2.2	1.5
Zn2Cys6	MGG_01518.6	TF169	1	1.2	1.7	2.2	1.7
Zn2Cys6	MGG_07063.6	TF230	1	0.9	1.4	2.3	1.6
Zn2Cys6	MGG_07131.6	TF231	1	0.4	0.6	2.0	0.6
Forkhead	MGG_06422.5	FOK1	1	0.1	0.9	0.2	2.2
GATA	MGG_02755.6	TF031	1	1.5	1.3	1.3	2.3
Myb	MGG_02746.6	TF046	1	0.8	1.3	1.6	2.2
C2H2	MGG_00702.6	TF070	1	1.8	1.6	1.9	2.2
C2H2	MGG_02474.6	TF116	1	0.4	0.5	0.6	2.1

C2H2	MGG_11252.6	TF133	1	0.6	0.7	0.7	2.5
Zn2Cys6	MGG_03711.6	TF192	1	0.6	0.6	0.7	2.1
Zn2Cys6	MGG_12037.6	TF264	1	0.7	1.0	1.3	7.6
Zn2Cys6	MGG_09825.6	TF268	1	0.1	0.2	0.2	2.3

: up-regulation (≥2)
