# Supplementary material for: Global Expression Profiling of Transcription Factor Genes Provides New Insights into Pathogenicity and Stress Responses in the Rice Blast Fungus
Source: PLoS Pathog. 2013 Jun 6;9(6):e1003350. doi: 10.1371/journal.ppat.1003350 (PMC3675110; doi:10.1371/journal.ppat.1003350)
Supplement: Table S7 — Primers used for quantitative real-time PCR. (PDF) [file ppat.1003350.s013.pdf]

Table S7. Primers used for quantitative real-time PCR

TF family	Gene locus version 6	Renamed gene TF	Renamed gene name	Forward primer (5'-3')	Reverse primer (5'-3')	Amplicon size (bp)
Forkhead	MGG_06422.5	FOK1		AAACGCAAGCGGTCTATCATGGAC	AGTTTGCTTTTAAGAAGGGGGGCC	155 bp
Forkhead	MGG_06258.5	FOK2		CAAGTCTCCAGACCAGAGTCCCA	TTGGAATCCCTTAGTGAGATCGAAGCC	196 bp
Forkhead	MGG_01853.5	FOK3		ACACTTTTGCTACACCCAGCAGC	ACTGCTGGCTGCTGGACAAAT	155 bp
Homeobox	MGG_04853.6	HOX1	*MoHOX1*	TTATCACGGCGGCGACTCG	TTGGTGGACGCTCTCGGGA	205 bp
Homeobox	MGG_00184.6	HOX2	*MoHOX2*	TGGGGTTCTGCAGCCATGTT	GTCCCGTGGTGTTACGTTCTGG	168 bp
Homeobox, C2H2, Homeodomain-like	MGG_01730.6	HOX3	*MoHOX3*	CTGATGTCGGGCATGCAGC	GCTGACTTGGACTCTGCACCAG	151 bp
Homeobox	MGG_06285.6	HOX4	*MoHOX4*	CGGTGGCTACTCTGGGGAT	CGCTTCCAGAAGGGTTGCTGC	168 bp
Homeobox	MGG_07437.6	HOX5	*MoHOX5*	CACGCTGCATCCCATCGGT	AGTGCGCAGTCCCATAAGGC	179 bp
Homeobox	MGG_11712.6	HOX6	*MoHOX6*	ACACGCGCGCTTATACTGCTC	ACCCACAACAGACTCATCGCC	170 bp
Homeobox	MGG_12865.6	HOX7	*MoHOX7*	CGGACGGCTCCAAGATTCTCC	CTGCCACGCTTCATGCCAA	142 bp
Homeobox, C2H2	MGG_12958.6	HOX8	*MoHOX8*	CGTCGTCACTCAACTCGGCT	CGGCATTGATGGGTTGAGGT	165 bp
bHLH	MGG_00595.6	TF001		GCCCTCACGCGGTCCTAAA	TGTAGGACGAGCAGTGGCAC	176 bp
bHLH	MGG_01090.6	TF002		ACGACCTAGTTTCCCAGCCG	ATCTGAGCGCCGGTTCCA	163 bp
bHLH	MGG_01321.6	TF003		CGGCAAGCGTCGTCTAACTGA	TGGGCCTCAGATCGTGACATTG	147 bp
bHLH	MGG_04359.6	TF005		CAATCCGGCCAAACGAATGACG	CGATGCCTCCACTGCTTTGTCT	135 bp
bHLH	MGG_05709.6	TF006		CCGCACCACACCGATTACAAAG	TACCGTCGTCGTCCTCGTTCC	169 bp
bHLH	MGG_10575.6	TF007		CCTGCGCACAAGCTCAACAAG	ACGGCCCCATTCATCACCAA	152 bp
bHLH	MGG_10837.6	TF008		GAACGGAGGCGACGGGTAT	CATTGGCTCTCGGCTGCTCA	134 bp
APSES	MGG_00692.6	TF009	*STUA*	GATGACCGTTCGAGCAGCAGTG	GCGGGGCGGTTCATTGTC	104 bp
APSES	MGG_08463.6	TF010	*MoAPS2*	CGGCACGGGCGAGAAGA	TTTACCATGCCATCCGACACCT	135 bp
APSES	MGG_09869.6	TF011	*MoAPS1*	AGCTGGTGCTAGTCCCTTCA	CCACCGTTAAATGTCTGGCT	297 bp
Zinc finger, GATA	MGG_01840.6	TF030		GGTGCGCCCAGAGGTGA	GAGCCGGGAGACAACGACG	125 bp
Zinc finger, GATA	MGG_02755.6	TF031		GATTCGGCACCACGGCTACG	AGTGCGCTTGTCGACATGCC	127 bp
Zinc finger, GATA	MGG_03538.6	TF032	*WC-1*	CAAGTTGGCCGAGTATCACCTC	GAGTTGTTTGCCGCGTTTCCATC	143 bp
Zinc finger, GATA	MGG_04521.6	TF033		CACTTGACTCTCCCGAATGGCG	GCTGCCGCTCCCTGTTT	119 bp
Zinc finger, GATA	MGG_06050.6	TF034		ATGGGCAAGAGGGTGGTGTC	GCTTCAGCCCTCAGTTGGTCT	166 bp
Zinc finger, GATA	MGG_07319.6	TF035		CCCGGACGATGCCACAGT	CTTTCTTGCTTGCCCCCGC	147 bp
Zinc finger, GATA	MGG_10970.6	TF036		CCCCACGTGGATGCTTCTCA	GGGACCTGCTGGACCGTT	105 bp
Myb, Homeodomain-like	MGG_00138.6	TF041		GTCCAAAGGGGCCACGAAAAG	CCACCAATCCCAACAAATCGACC	126 bp
Myb, Homeodomain-like	MGG_01012.6	TF042		CCACCCTGCTCACTACCATTGT	GCTGTCCTCGTCATTCTCCTCA	122 bp
Myb, Homeodomain-like	MGG_01133.6	TF043		CTACGCAGAATTCGCAGAGAACG	AGGGAACAGTTCGGGAAAGTGT	154 bp
Myb, Homeodomain-like	MGG_01426.6	TF044		AGACGTTGGCAGTTTCCGAGG	CCGACTCTTCTCTTATGCGCCTG	129 bp
Myb, Homeodomain-like	MGG_01720.6	TF045		GTCGGCTGCGGTCAATGTC	CTCAAGCTCCCGCCTCTCAG	100 bp
Myb, Homeodomain-like	MGG_02746.6	TF046		GAGACGAGCACAACGGGC	GCGTGTCGTCTAGAAGCAACCT	97 bp
Myb, Homeodomain-like	MGG_03899.6	TF047		GCCCAGCAATCGCCGTTG	CGTCTTGTCCGAGGCCGTG	162 bp
Myb, Homeodomain-like	MGG_05099.6	TF048		GCATCCAGCCGAAGCCTT	GCCTTGCCAACCCATCCAG	169 bp
Myb, Homeodomain-like	MGG_05240.6	TF049		GGGACACCACCGGATACACG	GCTGGGTGGGGTGGTAGATA	171 bp
Myb, Homeodomain-like	MGG_05748.6	TF050		GAGTGGAGCGCCGAGGA	CACGTCGACTCCTGCTGCTG	187 bp
Myb, Homeodomain-like	MGG_05945.6	TF051		CACTGGCACGCATCACTGG	GCCAATCCCCATCTCCACAC	194 bp
Myb, Homeodomain-like	MGG_06120.6	TF052		GGAGCTGGAGCGTGAGGAG	CCTCAACTGCAAGCCTCCTACG	182 bp
Myb, Homeodomain-like	MGG_06434.6	TF053		CAGCCCCCGACACCGAA	TCACTGCTGGCTTGTCGCTG	191 bp
Myb, Homeodomain-like	MGG_06898.6	TF054		CCAGATCCATCAACGCCGC	TCAAGCACGGAAGCGACTCG	170 bp
Myb, Homeodomain-like	MGG_08095.6	TF055		CCGAAAATGGACAGGCGACT	CACTACCCGCAGACAGCAC	156 bp
Myb	MGG_08137.6	TF056		GCTGGACGGCGGAAAACT	CGCCTCCAACCTTGTCCTTCA	144 bp
Myb, Homeodomain-like	MGG_10426.6	TF057		CTCTATCCAGCCGGCCGA	ATTTGACTGGGCAACTGGGC	120 bp
HMG	MGG_01171.6	TF059		GATGAGTTCGAGGGCGGGG	CTCCTTCGTCTGCTCCGGC	176 bp
HMG	MGG_07011.6	TF104		CGTGCCGGCCTATCTGCT	TTCGCTGTTGTCTCCATCCTCC	166 bp
Zinc finger, C2H2	MGG_00139.6	TF058		GCGGTGCTTCAGCTGAGGAT	TGTGCACGGTCATGACATCCATC	198 bp
Zinc finger, C2H2	MGG_03061.6	TF061		GCGCGAACCCAACGATACAC	CACCCGGAAGGCGTCTCT	86 bp
Zinc finger, C2H2	MGG_03520.6	TF062		CGAGTGCGCCAAGTGGTTC	CCACATCAGCATCGCCTCGT	179 bp
Zinc finger, C2H2	MGG_06690.6	TF063		GGCCCGATCATATCACCATCTGT	CTTGCCCTTGTCCGCATCTTTG	139 bp
Zinc finger, C2H2	MGG_07155.6	TF065		GACAAGGCCGGCATCACAC	GGTTCAGACCGAGACCACCAG	142 bp
Zinc finger, C2H2	MGG_08561.6	TF066		CCGAGTGATGGGGTTCAGCG	CTCCTGGGAATGGACACGCG	150 bp
Zinc finger, C2H2	MGG_09028.6	TF067		CATGGAGGAGCAAGGCAGTGG	CGGTCGGGTGTCGTGGAT	157 bp
Zinc finger, Rad18-type putative	MGG_09837.6	TF068		GGCCAGGCGGTTCGAAGA	TACCTCGCCGCACCTGG	153 bp
Zinc finger, C2H2	MGG_00076.6	TF069		ACGCATCCGACAACAAGCCG	CGCTGCTGCTGCTGATCA	166 bp
Zinc finger, C2H2	MGG_00702.6	TF070		TCCGGGGACAAGACGCAC	CTCGACGGTGCACGCAAC	160 bp
Zinc finger, C2H2	MGG_01586.6	TF071		AGATGGTATGCCCGGCGG	TCAGCTCACCGCCACCAC	182 bp
Zinc finger, C2H2	MGG_02447.6	TF073		CGTCTCGGCCGTCTTCTTCA	CTCCTCTATCCAGCCACCACC	197 bp
Zinc finger, C2H2	MGG_04546.6	TF075		CAGCGCGGGCCTCAAAG	CTACTGCAGCAAGACGTCCCT	151 bp
Zinc finger, C2H2	MGG_04580.6	TF076		GGAACAAGCGGGTCGAAGC	CATAATGTGACGACGCATGCCG	119 bp
Zinc finger, C2H2	MGG_04857.6	TF077		CCATAAGCGGGCAGTCAAGTCT	TATCGGCGTCCTCGTCCTGTAT	107 bp
Zinc finger, C2H2	MGG_05518.6	TF078		CGGGTAGCACTGTATACGCATGT	GCTATCGAATCTACGTTCATCGTGG	93 bp
Zinc finger, C2H2	MGG_06184.6	TF079		CACCTGCAGCTCCCAAACC	AGGTTTTGCGGGGGAATGTC	186 bp
Zinc finger, C2H2	MGG_06944.6	TF083		TTCAAGCGGCGCAAAGAAAGT	CTCTCTTGACCTCTTGCGCC	100 bp

Zinc finger, C2H2	MGG_07699.6	TF084		CATCAAGGGCGGCTCCTCA	CATTCGGAGTCGCTGTCGCTC	156 bp
Zinc finger, C2H2	MGG_08340.6	TF085		CAGCGTCCGCGGTCTCAAT	TCATCGTCGTCGTCATCTTCGTC	174 bp
Zinc finger, C2H2	MGG_09035.6	TF086		CCCTCAAACAGCGCTAGCAC	GCCTGAACCTCGCTGTAATTCG	134 bp
Zinc finger, C2H2	MGG_09700.6	TF087		CAAGTGCGGTGCGTCCATTG	ATGCGGATGCCCAGCCTT	195 bp
Zinc finger, C2H2	MGG_10162.6	TF088		CAGCCGGAGCAGGGTTCAT	CATCGTCGTCCATCGTACTATCAGC	146 bp
Zinc finger, C2H2	MGG_10276.6	TF089		CTACGGAGGAGCAGGAACAGG	CGCGGCTCCGTATTGATTCC	110 bp
Zinc finger, C2H2	MGG_10280.6	TF090		GCTGCGGCCCTGGTTC	CCCGTAGCGTGCCTCAAG	111 bp
Zinc finger, C2H2	MGG_00373.6	TF094		GCTCTATTGTGGCCCGTCTG	CGCTGTCTTCGTCGGCAC	183 bp
Zinc finger, C2H2	MGG_01776.6	TF097		ATCGGCCCTGGGTGCTCTA	AGTTATGCGCCCCTCTTTGGTT	113 bp
Zinc finger, C2H2	MGG_02036.6	TF098		GCGACAAAGAAATCCCTGACACGAC	GCAAACGGGCATTCCATTCTCCTT	114 bp
Zinc finger, C2H2	MGG_02775.6	TF100		GCTCAAGCGGCATCGACT	GCTTCATGTACGGGTAGTCGCC	181 bp
Zinc finger, C2H2	MGG_04699.6	TF101		GACCAAGTTCGCTCCAGACG	GTCCTCGGAGCCAGCCT	161 bp
Zinc finger, C2H2	MGG_05133.6	TF103	*MoCRZ1*	TCGGGCCGCAGTAGCTTTG	TTCGTTCATGCCGCCCTG	102 bp
Zinc finger, C2H2	MGG_07140.6	TF105		CAAGCTGGTGCGCAACAATCA	TGCATCACTCGGTCGGACG	117 bp
Zinc finger, BED-type & C2H2	MGG_08015.6	TF106		GCGGACTCCAAGGGCAAGAA	ACATGATGGGACCGCGGT	170 bp
Zn2Cys6, Zinc finger, C2H2	MGG_09780.6	TF109		CAGCAAGGGCGAGATGACGA	GTACGGCCTCAACCTCGC	193 bp
Zinc finger, C2H2	MGG_10150.6	TF110		TCAGTCGGATCGCCCTCG	TCTCTGTCTTGATCGGCGGC	181 bp
Zinc finger, C2H2	MGG_10664.6	TF111		ACTATATCGCCTCGCTCACATCAG	CACCAGGCCCCAACAGTC	176 bp
Zinc finger, C2H2	MGG_01017.6	TF113		GGCGGCGGCTAATGGAAG	GACTCCAGCCCATCGACCAT	148 bp
Zinc finger, C2H2	MGG_01209.6	TF114		CTGCGACCCTGAGCTCAAACT	CTCGTTGATGTTGGGCAGGC	142 bp
Zinc finger, C2H2	MGG_02474.6	TF116		GATGACGCGGCAAGTGAGG	TAGGTCGCGTGCTTGGAGAT	186 bp
Zinc finger, C2H2	MGG_02505.6	TF117		CTCCAACTACCGTGCCCTCA	CCTGGTGGTTGGCTGCCTT	170 bp
Zinc finger, C2H2	MGG_03581.6	TF118		GACAAGCAGCCCTGGAAGAGC	TTGGTGTGTTTCGGCGCC	124 bp
Zinc finger, C2H2	MGG_05955.6	TF121		TACACCCGCTCCGACAACCT	CTCGGGGACTTGGACACTCT	176 bp
Zinc finger, C2H2	MGG_06575.6	TF124		CCAAACCTCGCAAAGTCGCA	CATGTGAGTTGAGCGCGTGC	120 bp
Zinc finger, C2H2	MGG_06848.6	TF125		CGATGGCGAAGGAACAAACAGC	GCGCAGCAACCGACTCAG	144 bp
Zinc finger, C2H2	MGG_07269.6	TF127		AACCGGACCCAGCTTTTACCAT	GTGCGGGCATCGGAACT	134 bp
Zinc finger, C2H2	MGG_07314.6	TF128		ACGTTGGTCCAGTGGGCATC	GCATCCTCCTCACCGGCATT	161 bp
Zinc finger, C2H2	MGG_07339.6	TF129		GGATGAGGCGACCGAGAAGTTAG	CCGCCCCTGCTGCTGATA	132 bp
Zinc finger, C2H2	MGG_09701.6	TF131		GCATCTGACGGACGCACACA	ATCCATAGGCACATCCGGCTC	172 bp
Zinc finger, C2H2	MGG_00504.6	TF132		CGAGGGATGTGCACACTCCT	CTACTCCTTGAGCGCAGCCA	161 bp
Zinc finger, C2H2	MGG_11252.6	TF133		CGAGATCAACTGGCACGAGC	CCCCCAACAGAATCGGCATC	135 bp
Zinc finger, C2H2	MGG_02845.6	TF134		GCGTTGAAGGCTGCCCGTA	ATATGGGCATCGACCTCGGC	139 bp
Zinc finger, C2H2	MGG_03030.6	TF135		TACGGCCCCACTGATGATTTGA	GTTGACGTGCTTCTTGCTTACAGC	117 bp
Zinc finger, C2H2	MGG_03133.6	TF136		CGCTACCTCCTCTGACGATGC	TCAACGACCCCAGCCTTGTG	122 bp
Zinc finger, C2H2	MGG_04328.6	TF137		GCGCACCCGACAAAACGAC	TGGCCATCTGGAGCGAACAT	132 bp
Zinc finger, C2H2	MGG_04428.6	TF138		AGCAGAATTCACCCGCACCTC	CCTGGTACTGGTTCATGCGCC	114 bp
Zinc finger, C2H2	MGG_06328.6	TF139		ACGGCCTAGTGCACGAGTCA	ACCATGTCGTCTTCGCCGTC	197 bp
Zinc finger, C2H2	MGG_06364.6	TF140		CGCTCCGGCTCACGACTA	GCCACTCCTCCCACCTGT	140 bp
Zinc finger, C2H2	MGG_08493.6	TF141		CCATTTTTGCCGGACCCAGAGA	AGCGAGCATAACGTCCGAAACTT	179 bp
Zinc finger, C2H2	MGG_01127.6	TF142		AATGGCCGCAAGCAGAAGGT	CGACGTCGGGATGGCAAAC	106 bp
Zinc finger, C2H2	MGG_14806.6	TF143		GGCACAGTCCAATCGCTTATTCAG	CCAGTGGGTGTAGGCTGCT	150 bp
Zinc finger, C2H2	MGG_10595.6	TF146		CGACTTTGGCAGCGGATTGATGTA	CTTGTTGTTGCGGGATTCGTTGC	155 bp
Zinc finger, C2H2	MGG_14358.6	TF147		CCCAAATCAGCCCCGGTC	CTCCGCTCGCTGCTCACAA	133 bp
Zinc finger, C2H2	MGG_03977.6	TF148	*COS1*	GCCCGCCCGAAGTTACG	TCCGACGACGATGATGATGGC	144 bp
Zinc finger, C2H2	MGG_04456.6	TF149		GGCGACCGTCCGTTTTCTTG	AGAGGCCTTTCTTTGGTGTGGAC	188 bp
Zinc finger, C2H2	MGG_06507.6	TF150		TCGGTCGGATGGAGAGGC	ATGTGGACGTCGCTTCGGAGAT	177 bp
Zinc finger, C2H2	MGG_10764.6	TF151		GTAACCCACCACCGGCG	GCGCGGCTTGGTATCTGTC	187 bp
Zinc finger, C2H2	MGG_01987.6	TF152		GCATCATCGGGGACAGCATC	GAGCGGCAGGAGCATTGGTA	148 bp
Zinc finger, C2H2	MGG_05322.6	TF153		GGATGCAAAAGCCACCAAGAACC	CAGGGAACTCGCAGGGAAACT	174 bp
Zinc finger, C2H2	MGG_05714.6	TF154		CGGCAGCGCGATAACAAACC	CGTGGGCGGCATAGTAGTGG	118 bp
Zinc finger, C2H2	MGG_08114.6	TF155		CGGGCTGCCAGACCATTTTTG	GCCGGTACTCCCCATCCTC	171 bp
Zinc finger, C2H2	MGG_09200.6	TF156		CTCGCCCGGCACATCAAAC	CGTCGCGAGCCGTCAAAG	186 bp
Zn2Cys6	MGG_00021.6	TF158		ATCGAATGCGCACACTCCAG	GCCAGCAACGCCCTCAG	171 bp
Zn2Cys6	MGG_00049.6	TF159		CGAGCGCCGTGTCTGTTC	GCGTGGGCATCCTGGTC	195 bp
Zn2Cys6	MGG_00320.6	TF161		GCGGGAGTGGGCGAAC	GCGGCAGGCGTAAATGTAGATG	169 bp
Zn2Cys6	MGG_00494.6	TF164		CTGCTGCGAGAGACGTGGAC	AGGTAATCCCAGCCCTTTGTCTG	149 bp
Zn2Cys6	MGG_00672.6	TF165		AGGGAGGATTACGAAGCCAGG	TGTTTGCGGGTGCTGGTGTT	176 bp
Zn2Cys6	MGG_00934.6	TF166		ACGTCAACGGTCTCGCTGG	CATCCATGGCAGACACCTTGG	159 bp
Zn2Cys6	MGG_01285.6	TF167		CTGTGTGTTTGGGAGGAGATGCAG	ATGCGGGAAGCTTCTGGGAG	148 bp
Zn2Cys6	MGG_01414.6	TF168		CAGCATCCTCGAGTTCGACCC	ATGGTCTCGCATGCCTTGACC	147 bp
Zn2Cys6	MGG_01518.6	TF169		GCTATGCATCACAACGGCCACA	GGAACCATTCCTGACCGTCGAG	127 bp
Zn2Cys6	MGG_01624.6	TF170		CGGAAGCCAGTAGCGACGG	AAATTCGCCTGTCTGGGGGTC	161 bp
Zn2Cys6	MGG_01734.6	TF172		TGTCATGGGCGCAACTATGTCA	GCTCGCCTCCTGACCTCTG	183 bp
Zn2Cys6	MGG_01777.6	TF173		TGCCGCCCCAACTTCATGAG	GGATGGGTGTCGTGGTGGAT	155 bp
Zn2Cys6	MGG_01833.6	TF175		ATGGGAATGATGGGGGTCTCG	GTCGTGGCTGGGATGTCGT	161 bp
Zn2Cys6	MGG_11119.6	TF176		GCAGGACATTTGGCCGCTC	GCGGCTGGCCAAAGTTCA	125 bp
Zn2Cys6	MGG_01946.6	TF178		AATCCCCAAAGGGAGCCAGTG	GACACAGGTCCATAAGTTGCACCAT	161 bp
Zn2Cys6	MGG_02377.6	TF180		ACTGAGTCCCGCGTCTGC	GCTGATCCTCGACTGGCTCT	103 bp

Zn2Cys6	MGG_02408.6	TF181	AGCGCCAAGGGACATCACG	GCCAGCTGCCCTATCCCAC	164 bp
Zn2Cys6	MGG_02595.6	TF182	GGAGAGCTGGTGACGAGACG	CCCAAATCCTCCCCAGCCG	160 bp
Zn2Cys6	MGG_02600.6	TF183	CGGGGTGGGATTGCGACTT	TCTCGTCGTGATCCCAAAGCAG	171 bp
Zn2Cys6	MGG_15023.6	TF184	AGCACACGAGCCTTTCACGC	GCACAACCACGCCGAGGAAT	196 bp
Zn2Cys6	MGG_15021.6	TF185	ACCAGAGCATCGAGAGACGG	GCACGTTAGAGTCCAATCCGCC	164 bp
Zn2Cys6	MGG_02880.6	TF186	GACCTAATCAGTCGGTCGAGCAG	TAGAGCCGCGAACGCTGTAC	138 bp
Zn2Cys6	MGG_03055.6	TF187	CGTTGGATAACTACATCCCGGTGTG	CCGTCCATACCGCTGTCTAGATCA	139 bp
Zn2Cys6	MGG_03183.6	TF188	CCGACCACGAGGTCTACAACATTAG	TGCGCCTGACAGGCTCAAAG	150 bp
Zn2Cys6	MGG_03463.6	TF190	TCCAAACCGCCTCGAGAGTG	CAAATCGAGTCCAACCGGGC	177 bp
Zn2Cys6	MGG_03711.6	TF192	ATTCGACGGAGCAGCTGAGG	TCACCCATGCCGCCCATC	186 bp
Zn2Cys6	MGG_03763.6	TF193	CTCGTGGATCGGATCTTTTCGCC	TGCGACAGACCAACCAGCT	125 bp
Zn2Cys6	MGG_03848.6	TF194	ACACCTGCACCACAAATGATGGAC	CAAAGTCGTCGTCTCCGCTGA	139 bp
Zn2Cys6	MGG_03939.6	TF195	ACGCCGAACGGTGGATACTA	GCCCCCGTTGTTCTCTTCGC	177 bp
Zn2Cys6	MGG_04108.6	TF196	AACCTCAGGTGGGTGGTCAC	CGTCACTGATGCTCAAGACGCTTTG	125 bp
Zn2Cys6	MGG_04213.6	TF198	CACGATGGCTGGGACACAAG	TCATATCATCTGCCCCGCGT	175 bp
Zn2Cys6	MGG_14900.6	TF199	AGTCAACACCAGGCTCAGCA	TCAGCCAAGAGATGTCCCGTC	153 bp
Zn2Cys6	MGG_04326.6	TF200	AACTGCGGGCGATTGGAACC	CCACCACTGCTCGACACCT	173 bp
Zn2Cys6	MGG_04387.6	TF202	AGGGGTCTCTGTCCATGGCT	TCATGGCACAAAGCGTTCGC	140 bp
Zn2Cys6	MGG_04571.6	TF203	GAGGTGATTTGGTCGTCGGC	GATGAGGAGCCGTCGTGTGC	178 bp
Zn2Cys6	MGG_04674.6	TF205	CGAGCAGTTCATGGACCAGC	CCTTGCCATCAGCCTCCC	172 bp
Zn2Cys6	MGG_04951.6	TF206	GGGCAGATTCAGCGCGTG	TCACAAAACTCCGCCCTTGG	132 bp
Zn2Cys6	MGG_04970.6	TF207	GCAGAGCCGCGATCGAGAC	TTCAAGCTCCACCACCACCG	148 bp
Zn2Cys6	MGG_15139.6	TF209	TTCCGCGCTGAAAGTCGC	ATCGCTCGTCACGTCACGTTTTG	126 bp
Zn2Cys6	MGG_05343.6	TF210	TGCCTCCCCCATCGAACAAT	GTGCCCATCATACCTCCGCC	131 bp
Zn2Cys6	MGG_05578.6	TF213	ATGCAGCGACCATGTACAGGAT	CTTACTCTCTTGTCGTCCGCCG	118 bp
Zn2Cys6	MGG_05829.6	TF215	CGGGTCATGATGACGGAGGAT	CGACAGCACGCTGTCCAT	129 bp
Zn2Cys6	MGG_05845.6	TF216	AGATGCTGCAGAGGATCGAGG	AAGTCCGAAGCAGTGCGGG	144 bp
Zn2Cys6	MGG_05891.6	TF217	TTGAGCATGGCTGGCGC	CGCCGTCCAAGACTCAAACAG	132 bp
Zn2Cys6	MGG_05939.6	TF218	CCGTGAACAATCCGGTTGGGATC	GGCAGCTCGATCCCCATGA	133 bp
Zn2Cys6	MGG_06086.6	TF219	GTCTTTGAGCTCGACGCCAGAG	CTCCCATCTTCTTGCTCGCCAG	128 bp
Zn2Cys6	MGG_06243.6	TF220	GGTATGACAAGCCAGATGCTGGAT	GGCCGCGTGCAGTACTT	117 bp
Zn2Cys6	MGG_06279.6	TF221	AACTCGTCGTGCAGCTCG	TCGTCGGCAAGACCTGCAG	152 bp
Zn2Cys6	MGG_06355.6	TF223	ATGGATGGCTTTGGCAACTTTGGAT	GCCTGTGCTGAGTTGGTCATGA	139 bp
Zn2Cys6	MGG_06492.6	TF226	ACTGAACAAGCTCCCCGAGAAG	TAGGCTGCCAGCCACACC	142 bp
Zn2Cys6	MGG_06626.6	TF227	GCACTACCAAGAACACGACCG	TTCAAGTACTTGCCATAGGCGGC	141 bp
Zn2Cys6	MGG_06832.6	TF228	CCTGTAGATGGATAGAGTCTCCCCC	CGCTTTCCCCATTCTCATTCGTCT	132 bp
Zn2Cys6	MGG_06954.6	TF229	TGATCGCGATACTGCGCG	CCCCTCACAGTCTCTTCAGTTCC	135 bp
Zn2Cys6	MGG_07063.6	TF230	TACCTGCTTCTAGGAAGCCGTCC	CTTCTTGCTCTTACGGCATCGCT	119 bp
Zn2Cys6	MGG_07131.6	TF231	GGTACAGTATCAAGATAACGCCCAGAGA	CTTGATCCAGGCCGATTCGCAA	124 bp
Zn2Cys6	MGG_07215.6	TF232	TCAGGCTTGTTCGATCGATGCC	TGTCCTCGCGCTTGAGGG	129 bp
Zn2Cys6	MGG_07218.6	TF233	ATCAACAGTATGAGCGGCAACCTG	TTCCAGTTTGCGCAGCTCC	135 bp
Zn2Cys6	MGG_15085.6	TF234	AAAGACCGCAGATCCTTCAGGAA	ATCTTCGTCTGCCACACTGCC	148 bp
Zn2Cys6	MGG_07368.6	TF235	ACTTCAAGGGCATCCTGTGGC	GTGCAGCAGGCCGTACAT	110 bp
Zn2Cys6	MGG_07458.6	TF237	AGGTCGTCTATTACGCTACCAGCC	ATGATATCGCGGCACTTGGACC	139 bp
Zn2Cys6	MGG_07534.6	TF238	GCGTTCTGATGTACATTGGGTGGG	ATCAGGAACTCTTGAAAGGCAGGC	136 bp
Zn2Cys6	MGG_07549.6	TF239	AGAAGCTGTACGACATTGCCTGC	ACGAGGCCCCAGACAAAACG	124 bp
Zn2Cys6	MGG_07681.6	TF241	GCCGCTCGTCGATCCTTCTA	TGTTGCTGTTGACCAGGGGG	144 bp
Zn2Cys6	MGG_07777.6	TF242	GGTAGGGAGCCAGGTGACG	GGACCCCCAACGTCTCTTGT	168 bp
Zn2Cys6	MGG_07845.6	TF244	GAAGTGCGCTGGTGCTGG	CCAGATGCTGCTGCAGATGC	150 bp
Zn2Cys6	MGG_08094.6	TF246	GCCGTTGGACGATGACTTCC	TCAAGCGTTGACTGGCGATCT	115 bp
Zn2Cys6	MGG_08168.6	TF247	CGGGTCGATTTCAGCCTCGG	TCCCGGATACAGCCCTCTGG	145 bp
Zn2Cys6	MGG_08185.6	TF248	ACCACCAACACCCTCACCC	TACCGTATCCTGGGCAGCTC	175 bp
Zn2Cys6	MGG_08199.6	TF249	AATTCCCCGCTGATGGTGCC	ACCTGCTGTGCGTTATTCCCA	142 bp
Zn2Cys6	MGG_08314.6	TF250	AATGCGCACGGGAACGGA	GCCAATCCCTGTTGACGTCC	136 bp
Zn2Cys6	MGG_08361.6	TF251	CCTTTCGGGGCCCTTGATGA	TCACCAGGCGTGCTCGTC	130 bp
Zn2Cys6	MGG_15346.6	TF253	ACGGGGAGGGAAGGGGATTT	GCTATACCCGAGCCGTCTGG	167 bp
Zn2Cys6	MGG_08753.6	TF255	AGTAGAGGTGGCGTCTGCG	ACACACCGTCTAGCCGTACA	122 bp
Zn2Cys6	MGG_08917.6	TF257	CCAGGAGATCTACCGCGCAA	TCAAGGCCGAGTGACCTGC	154 bp
Zn2Cys6	MGG_09263.6	TF260	CCCATCCCGATTTCCACCAC	ACATCCACTGCTCCGTTGGG	163 bp
Zn2Cys6	MGG_09276.6	TF262	CGTCCTGCCCGGCATGAT	CATGCCGGCCGAGTCCAATA	160 bp
Zn2Cys6	MGG_09312.6	TF263	AACGTCGCCGCACTGGAG	TATCCCTCAACCCGACCTGGC	160 bp
Zn2Cys6	MGG_12037.6	TF264	TCGGCTTTAGTGGTGCGGAG	AATGCGGACCCGTTGAAGC	160 bp
Zn2Cys6	MGG_09676.6	TF265	AGGAGCTGTGGGACCTGG	AAAGCATGCAGCTCCTCCAG	193 bp
Zn2Cys6	MGG_09825.6	TF268	CCCCGATCCAAATGACGCC	AGGAATTAGGTGCCACCCCG	185 bp
Zn2Cys6	MGG_09829.6	TF269	CGTGGGCATTTCCGACCATT	TAATGCCGCTGCGAACTGTC	193 bp
Zn2Cys6	MGG_09950.6	TF271	AGCAGAAGTACAGCGGCAGC	ACTACGAAGCTGCGCGAGAA	176 bp
Zn2Cys6	MGG_10197.6	TF272	TAGCTCACGGGCATGCTCTG	CACTCCAGCCCGGCCTTTAT	157 bp
Zn2Cys6	MGG_10307.6	TF273	GAGGAGGAGCTGAGAAGGCAT	GTAGAATGCCGCTGCGCCTT	169 bp
Zn2Cys6, HMG	MGG_10422.6	TF275	GTCCACAGGGTCAAGGGCTA	CGAGGCCCATCCTGTATGCG	142 bp

Zn2Cys6	MGG_10528.6	TF276	CCCAATGGACCCGATGAGGC	CCTCACTTCATCTGGGGCAC	160 bp
Zn2Cys6	MGG_10802.6	TF278	ATGAAGACCCGTACGCAGCG	CAAACTCGTCCAAGCCGGC	180 bp
Zn2Cys6	MGG_10806.6	TF279	GCGCTTGCTGGACAGTGC	TCAGCCAGTGCTTCTACCGC	142 bp
